# Integrating network pharmacology, molecular docking and dynamics simulation to decipher the antipyretic mechanisms of Xiaochaihu granules

**DOI:** 10.3389/fmed.2026.1772991

**Published:** 2026-02-20

**Authors:** Minghe Gu, Hong Liu, Cong Bi, Wenhui Situ, Haiyong Du, Aihua Lin, Junhua Zhang, Yiming Liu

**Affiliations:** 1 The Second Clinical College of Guangzhou University of Chinese Medicine, Guangzhou, China; 2 Guangzhou Baiyunshan Guanghua Pharmaceutical Co., Ltd., Guangzhou, China; 3 Guangdong Provincial Hospital of Chinese Medicine-Zhuhai Hospital, Zhuhai, China; 4 Guangdong Provincial Key Laboratory of Clinical Research on Traditional Chinese Medicine Syndrome, Guangzhou, China

**Keywords:** experimental validation, fever, molecular docking, molecular dynamics simulation, network pharmacology, Xiaochaihu granules

## Abstract

**Background:**

Xiao-Chai-Hu granules (XCHG), a classical traditional Chinese medicine formula derived from the ancient text Erta Treatise on Febrile Diseases, has demonstrated established clinical efficacy in fever management; however, the underlying antipyretic mechanism remains incompletely understood.

**Methods:**

This study employed an integrated computational-experimental approach combining network pharmacology, molecular docking, molecular dynamics (MD) simulation, and cellular validation to systematically elucidate XCHG’s mechanism of action. Functional validation was performed in lipopolysaccharide (LPS)-stimulated RAW264.7 macrophages using nitric oxide (NO) assay, enzyme-linked immunosorbent assay (ELISA), quantitative real-time polymerase chain reaction (qRT-PCR), and Western blot analysis.

**Results:**

Through analysis of 18 pharmacokinetically validated blood-absorbed components, we identified 120 fever-related targets, from which 17 core targets and 5 key bioactive compounds (Oroxylin A, Wogonin, Baicalein, Liquiritigenin, and Enoxolone) were screened. Gene Ontology (GO) and Kyoto Encyclopedia of Genes and Genomes (KEGG) enrichment analyses revealed that XCHG modulates inflammation, immune regulation, and key signaling pathways including PI3K-Akt, MAPK, and EGFR tyrosine kinase inhibitor resistance. Molecular docking identified three high-affinity component-target pairs: EGFR-Enoxolone (−9.3 kcal/mol), ESR1-Liquiritigenin (−8.7 kcal/mol), and SRC-Baicalein (−8.4 kcal/mol), with 100-ns MD simulations confirming the structural stability and binding persistence of these complexes. In LPS-stimulated RAW264.7 macrophages, XCHG dose-dependently inhibited NO production and suppressed pro-inflammatory mediators (TNF-*α*, IL-6, IL-1β, PGE2) and enzymes (iNOS, COX-2). Western blot analysis provided direct target validation, demonstrating that XCHG attenuates p-EGFR and p-SRC phosphorylation while restoring ESR1 expression.

**Conclusion:**

Mechanistically, XCHG exerts comprehensive intervention across the inflammatory-pyrogenic axis through a dual mechanism: upstream blockade of EGFR-SRC signaling coupled with ESR1-mediated immune homeostasis restoration, distinguishing it from conventional single-target antipyretics. This study provides systematic mechanistic insights supporting the evidence-based clinical application of XCHG and establishes a replicable methodological framework for investigating complex herbal formulas.

## 1 Introduction

Fever, a hallmark of infectious and inflammatory diseases, can lead to serious complications including febrile seizures, dehydration, and multi-organ damage (1, 2). As one of the most common reasons for pediatric emergency visits, fever represents a substantial healthcare burden (3), making effective and safe antipyretic treatments a critical clinical need. The pathophysiology of fever involves complex immune-neuroendocrine interactions. Pathogen-associated molecular patterns activate immune cells to release pro-inflammatory cytokines (IL-1β, IL-6, TNF-*α*), which in turn trigger cyclooxygenase-2 (COX-2)-mediated prostaglandin E2 (PGE2) synthesis in hypothalamic endothelial cells, ultimately resetting the thermoregulatory set-point (4, 5). Although non-steroidal anti-inflammatory drugs (NSAIDs) targeting COX enzymes are widely used, they are associated with gastrointestinal complications in 15–30% of users, as well as cardiovascular risks and potential immunosuppression (6–8). These safety concerns underscore the need for alternative therapeutic strategies, particularly for vulnerable populations such as children and elderly patients.

Xiao-Chai-Hu granules (XCHG), a classical Traditional Chinese Medicine formula derived from Xiao-Chai-Hu-Tang, was first documented in the *Treatise on Febrile Diseases* (*Shang Han Lun*) by Zhang Zhongjing approximately 1,800 years ago (9). Clinical studies and systematic reviews have demonstrated its efficacy in managing fever across diverse etiologies. A systematic review and meta-analysis of 18 randomized controlled trials involving 1,424 patients showed that XCHG significantly accelerates temperature normalization, reducing the mean time to defervescence by 5.29 days (MD = −5.29, 95% CI: −5.59 to −4.99) compared to conventional therapies (10). Moreover, XCHG exhibits a favorable safety profile, with an adverse event rate of 8.86% when used as monotherapy—significantly lower than that of conventional Western medicine treatments—and no serious adverse events reported (10, 11).

The formula comprises seven herbs: Bupleurum chinense, Scutellaria baicalensis, *Pinellia ternata*, *Codonopsis pilosula*, Glycyrrhiza uralensis, *Zingiber officinale*, and *Ziziphus jujuba*. Previous pharmacological studies have identified the bioactive compounds and their anti-inflammatory activities from these individual herbs. Saikosaponins derived from Bupleurum chinense inhibit the production of PGE2, TNF-*α*, and IL-1β during inflammatory processes (12), Glycyrrhizic acid from Glycyrrhiza uralensis regulates the inflammatory process through the activation of glucocorticoid receptors and the PI3K/AKT/GSK3β pathway (13). Baicalein from Scutellaria baicalensis reduces COX-2 expression and PGE2 synthesis (14). Additionally, evidence suggests synergistic interactions among XCHG components that enhance therapeutic effects beyond individual compounds (15). However, these studies predominantly focused on isolated compounds or single targets, failing to capture the multi-component, multi-target synergistic nature characteristic of traditional herbal formulas.

Despite accumulating clinical evidence and component-level pharmacological research, critical knowledge gaps impede comprehensive mechanistic understanding of XCHG’s antipyretic action. First, the specific bioavailable compounds actually absorbed into systemic circulation and reaching fever-regulatory sites have not been systematically identified through pharmacokinetic profiling. Second, the comprehensive network of component-target-pathway interactions underlying fever reduction has not been constructed, limiting insight into how XCHG achieves therapeutic effects through synergistic multi-level regulation. Third, functional validation in relevant cellular models directly linking predicted molecular targets to antipyretic phenotypic outcomes is lacking. Addressing these gaps is essential not only for mechanistic understanding but also for evidence-based clinical optimization and potential new drug development from XCHG.

Network pharmacology combined with molecular docking and molecular dynamics (MD) simulation offers a powerful integrative approach to elucidate the complex mechanisms of multi-component herbal medicines (16). This systems biology-based method has successfully revealed the hepatoprotective mechanisms of berberine (17), artemisinin derivatives in inflammation (18), and other traditional medicines (19, 20), bridging the gap between traditional empirical use and modern pharmacological understanding. MD simulations, which model protein-ligand interactions in physiological conditions over time, demonstrate high accuracy in predicting experimental binding affinity and have become gold-standard tools for validating computational predictions (21). However, despite its proven utility, this comprehensive computational-experimental approach has not been systematically applied to investigate XCHG’s antipyretic mechanisms, representing a significant opportunity for mechanistic discovery.

**Figure 1:**
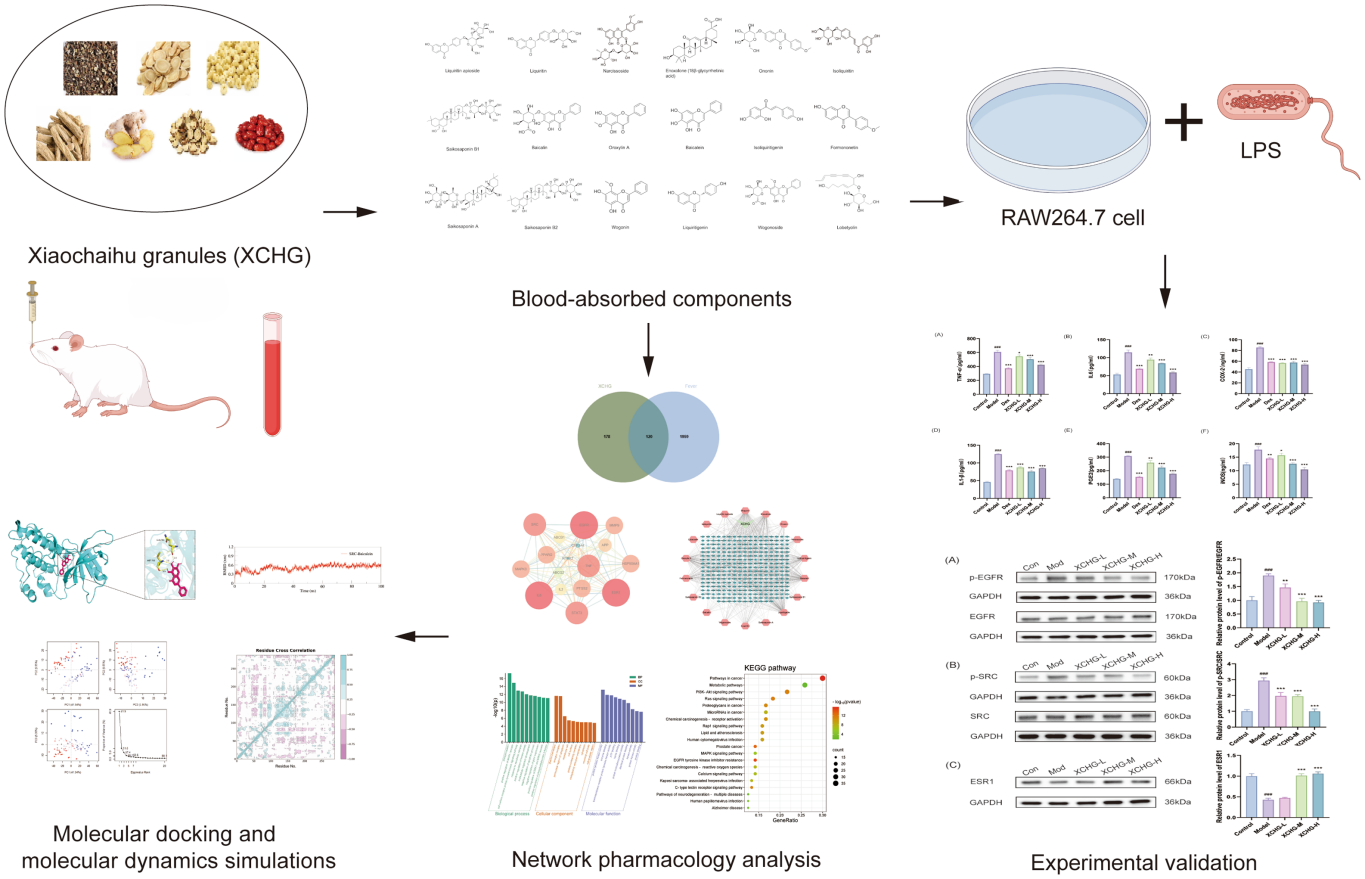
The technical workflow of the study. Created with Figdraw (www.figdraw.com), reproduced with permission (ID: TOTYR1d3a3).

The present study aims to address these knowledge gaps through three integrated objectives: (1) identify antipyretic-related protein targets of blood-absorbed bioactive components from XCHG through integrated database mining and network analysis; (2) construct and systematically analyze the component-target-pathway network to reveal multi-level regulatory mechanisms governing fever reduction; and (3) validate key component-target interactions through molecular docking and MD simulation, with functional confirmation at both the protein and phenotypic levels. This research provides the first systematic, multi-level investigation of XCHG’s antipyretic mechanism through integrated computational and experimental validation, including molecular-level target expression verification and cellular functional assays, offering mechanistic insights that support evidence-based clinical application while establishing a replicable methodological framework for studying complex herbal formulas. The technical workflow is illustrated in Figure 1.

## 2 Materials and methods

**Figure 2:**
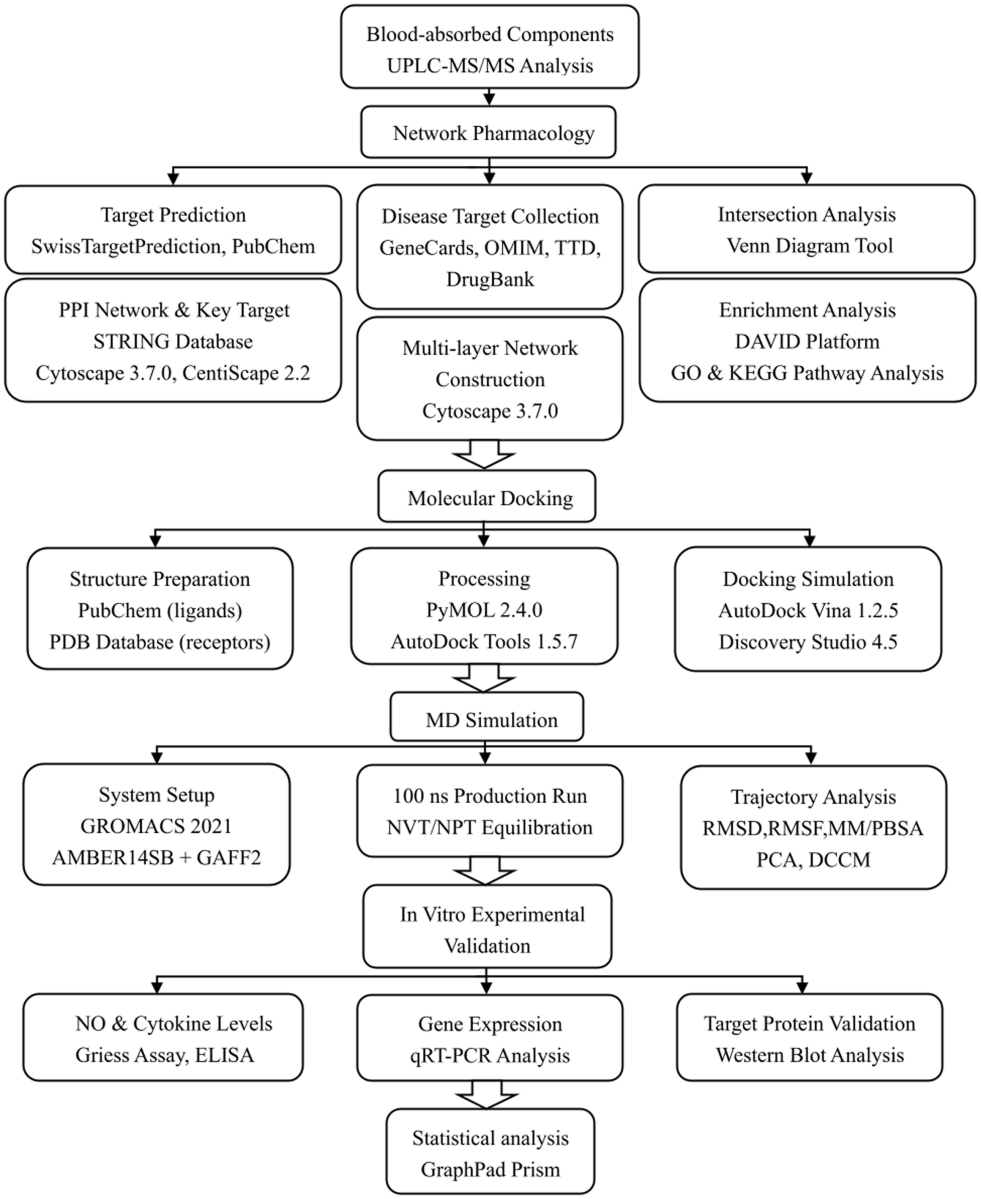
Flowchart of the methodological framework and analytical tools used in this study.

To improve clarity and make the methodology easier to follow, a flow diagram outlining the methodological framework and specifying the tools used in each step is presented in Figure 2.

### 2.1 Collection of targets for blood-absorbed components of XCHG

**Figure 3:**
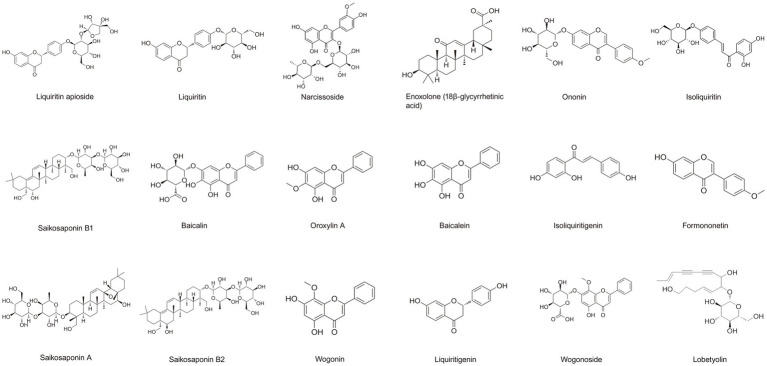
Chemical structures of 18 XCHG-absorbed components identified in rat plasma.

The blood-absorbed components used in this study were derived from our previous work (22), which systematically identified the prototype components absorbed into rat plasma after oral administration of XCHG. Briefly, in that study, a rat fever model was established by subcutaneous injection of 20% dry yeast suspension, and both normal and model rats were orally administered XCHG at a dose of 12.96 g·kg^−1^. Blood samples were collected from the orbital vein at multiple time points (0.25, 0.5, 1, 2, 4, 6, and 8 h) after administration. Plasma samples were processed by protein precipitation with acetonitrile and analyzed using ultra-high performance liquid chromatography coupled with triple quadrupole mass spectrometry (UPLC-MS/MS) in multiple reaction monitoring (MRM) mode. Through comparison of reference standards, blank plasma, and plasma samples from both groups, 18 prototype blood-absorbed components were identified (Figure 3).

The SMILES structures of these 18 components were retrieved from the PubChem database^1^ and then submitted to SwissTargetPrediction^2^ for target prediction, with the species parameter set to *Homo sapiens*. After merging and removing duplicates, we obtained the potential targets of XCHG’s blood-absorbed components.

### 2.2 Collection of fever-related targets

Fever-associated targets were systematically retrieved from four major databases: OMIM^3^, TTD^4^, DrugBank^5^, and GeneCards^6^. After merging non-redundant entries, we cross-referenced these with XCHG’s blood-absorbed component targets to identify potential anti-pyretic candidates. Target overlaps were visualized via a Venn diagram tool^7^.

### 2.3 PPI network construction and key target screening

Intersection targets were analyzed using the STRING database^8^ under *Homo sapiens* settings (interaction skey ≥ 0.4; free proteins hidden). The resulting interactions (TSV format) were imported into Cytoscape 3.7.0 for visualization and topological assessment. Network centrality parameters—degree (DC), betweenness (BC), and closeness centrality (CC)—were computed via the CentiScape 2.2 plugin. Targets with DC, BC, and CC values exceeding their respective means were defined as key targets, and a condensed PPI subnetwork was generated. Node properties (size/color intensity) in the visualization reflect interaction degrees, with larger, darker nodes indicating higher connectivity. Final topological validation was performed using Cytoscape’s ‘Network Analyzer’ plugin.

### 2.4 Construction of the XCHG’S ‘herb-component-target-fever’ network and screening of key components

To elucidate the multi-scale therapeutic mechanisms of XCHG, we first constructed a blood-absorbed component-target network of XCHG using Cytoscape 3.7.0 to identify pharmacologically relevant interactions between bioavailable components and their molecular targets. Building upon this foundation, we further constructed an integrated “herb-component-target-fever” network by mapping herbal components to their shared targets with fever-related genes. This comprehensive network integrates multilayered information, including herbal sources, bioactive components, molecular targets, and fever-associated pathways. Topological analysis using the Network Analyzer plugin identified five key components exhibiting the highest degree and betweenness centrality values, indicating their pivotal roles in mediating XCHG’s antipyretic effects through multi-target modulation of fever-associated pathways.

### 2.5 Functional enrichment analysis

Functional enrichment analysis of XCHG’s fever-related targets was performed using the DAVID platform^9^, encompassing Gene Ontology (GO) terms and KEGG pathways (23). Significant results were visualized as bubble plots and bar charts through the bioinformatics online platform (See Footnote text 7), highlighting key biological processes and signaling pathways modulated by XCHG.

### 2.6 Molecular docking

To investigate the interaction mechanisms between key components of XCHG and their corresponding targets, molecular docking analysis was performed. The five key components were selected as ligands, and the six highest-degree targets from the network were used as receptors. Component structures were obtained from the PubChem database, while target protein structures were retrieved from the Protein Data Bank (PDB) using the following criteria: human origin (*Homo sapiens*), X-ray diffraction resolution ≤3 Å, and publications within the last decade. Molecular docking was performed using AutoDock Vina (version 1.2.5) and AutoDock Tools (version 1.5.7). Protein structures were prepared using PyMOL (version 2.4.0) to remove water molecules, original ligands, and heteroatoms, followed by addition of polar hydrogen atoms and assignment of Kollman charges in AutoDock Tools. Ligand structures were processed by adding hydrogen atoms, optimizing geometric conformations, assigning Gasteiger charges, and converting to PDBQT format. Docking calculations were conducted with exhaustiveness = 24 and number of binding modes = 9. Binding site definitions and docking box parameters are provided in Supplementary Table S1. Each docking calculation was performed in multiple replicates to ensure reproducibility, with statistical analysis presented in Supplementary Table S2. All docked complexes were visualized using PyMOL (Supplementary Figure S1), and the top three complexes were further analyzed using Discovery Studio 4.5 Client to generate 2D interaction maps.

### 2.7 Molecular dynamics simulation

Molecular dynamics (MD) simulations were performed using GROMACS 2021 to evaluate the structural dynamics and binding characteristics of the three most stable complexes identified from docking studies. Systems were parameterized using the AMBER14SB force field for proteins and GAFF2 for ligands, solvated with the SPC/E water model in periodic cubic boxes extending 1.2 nm from the complex surface, and neutralized with NaCl. Long-range electrostatic interactions were calculated using the particle mesh Ewald (PME) method. Prior to production runs, systems underwent energy minimization using the steepest descent algorithm (50,000 steps), followed by NVT and NPT equilibration (50,000 steps each at 310 K and 1 atm with a 2 fs timestep). Production simulations were conducted for 100 ns with LINCS constraints applied to hydrogen bonds, saving coordinates every 10 ps. Each simulation was performed in triplicate to ensure reproducibility, with statistical analysis presented in Supplementary Table S3. Binding free energies were calculated using the MM/PBSA method. Trajectory analysis included root-mean-square deviation (RMSD), root-mean-square fluctuation (RMSF), radius of gyration (Rg), hydrogen bond formation, and solvent-accessible surface area (SASA). Principal component analysis (PCA) and dynamic cross-correlation matrices (DCCM) were employed to characterize collective motions. Free energy landscape (FEL) construction and residue-specific energy decomposition were performed, with conformational evolution monitored at 25-ns intervals throughout the simulation.

### 2.8 Materials

The commercial preparation utilized in this study was manufactured by Guangzhou Guanghua Pharmaceutical Co., Ltd. The composition of the granules consists of seven medicinal herbs in specific proportions: Bupleurum chinense DC. [Apiaceae] roots (Chaihu), Scutellaria baicalensis Georgi [Lamiaceae] roots (Huangqin), ginger-processed *Pinellia ternata* [Araceae] tubers (Jiangbanxia), *Codonopsis pilosula* [Campanulaceae] roots (Dangshen), fresh *Zingiber officinale* [Zingiberaceae] rhizomes (Shengjiang), Glycyrrhiza uralensis [Fabaceae] roots/rhizomes (Gancao), and *Ziziphus jujuba* [Rhamnaceae] fruits (Dazao). The crude drug-to-granule ratio is 0.486:1 (w/w), indicating that each gram of granules contains the equivalent of 0.486 g of raw herbal materials. RAW264.7 murine macrophages and DMEM complete medium were sourced from Wuhan Pricella Biotechnology Co., Ltd. (Wuhan, China), while LPS was acquired from Sigma-Aldrich (USA). Reagents including the NO assay kit and CCK-8 came from Beyotime Biotechnology (Shanghai, China), with ELISA kits for TNF-*α*, IL-6, COX-2, IL-1β, PGE2, and iNOS provided by Jiangsu Meimian Industrial Co., Ltd. (Jiangsu, China). Primary antibodies against p-EGFR, EGFR, p-SRC, SRC, and ESR1 were purchased from Abcam (Cambridge, UK). All primers were commercially synthesized by Genewiz Co., Ltd. (Suzhou, China).

### 2.9 Cell culture

RAW264.7 cells were seeded in DMEM supplemented with 10% FBS, 100 IU/mL penicillin, and 100 IU/mL streptomycin, cultured at 37 °C in 5% CO₂ for 1–2 days. When confluence reached about 80%, cells were passaged. Cells in the logarithmic growth phase were used for experiments.

### 2.10 Cell viability assay

RAW264.7 cells at 80–90% confluence were seeded in 96-well plates at a density of 1 × 10^4^ cells/well (100 μL/well) and allowed to adhere for 24 h at 37 °C under 5% CO₂. After aspiration of the supernatant, treatments were initiated. XCHG was directly dissolved in complete culture medium (RPMI 1640 supplemented with 10% FBS, 100 IU/mL penicillin, and 100 IU/mL streptomycin), followed by sterile filtration through 0.22 μm PVDF membranes. Experimental groups received 100 μL of XCHG-containing medium at concentrations of 0.5, 1, 1.5, 2, 2.5, and 3 mg/mL, while control groups were treated with equal volumes of freshly prepared medium without XCHG. Following 24 h incubation under identical conditions, the medium was aspirated and replaced with 10 μL CCK-8 solution. After 2 h incubation at 37 °C/5% CO₂, absorbance was measured at 450 nm using a microplate reader to determine cell viability.

### 2.11 NO release assay

RAW264.7 cells were plated in 96-well plates at a density of 1 × 10^4^ cells per well. The experiment comprised six groups: control, model, positive control (10 μM dexamethasone (Dex)), and XCHG-treated groups (low, medium, and high doses: 1, 1.5, and 2 mg/mL, respectively). After 24 h of culture, except for the control group, all groups were stimulated with LPS(1 μg/mL) and treated with corresponding drug concentrations for 24 h. Following 24 h of incubation at 37 °C with 5% CO₂, supernatants were collected. The supernatant was mixed with Griess reagent and incubated at room temperature for 5 min. Absorbance was measured at 540 nm using a microplate reader. A standard curve was generated using sodium nitrite standards, and NO concentrations in test samples were calculated based on their absorbance values.

### 2.12 ELISA assay

Cells were seeded in 6-well plates at a density of 3 × 10^5^ cells/well and divided into the following groups: blank control, model, positive control (10 μM Dex), and XCHG-treated groups (low, medium, and high doses: 1, 1.5, and 2 mg/mL, respectively). After 24 h of culture, except for the control group, all groups were stimulated with LPS and treated with corresponding drug concentrations for 24 h. The cell supernatants were then collected, and the concentrations of TNF-*α*, IL-6, COX-2, IL-1β, PGE2, and iNOS were determined using ELISA kits according to the manufacturer’s instructions. The absorbance was measured at 450 nm, and the cytokine concentrations were calculated based on standard curves.

### 2.13 qRT-PCR analysis

The culture grouping conditions of RAW264.7 cells were performed according to Section 2.12. Total RNA was isolated from the cells using Trizol reagent, followed by reverse transcription with a cDNA synthesis kit. For the quantification of TNF-α and IL-6 genes, qRT-PCR amplification was carried out under specific conditions. Initially, there was a denaturation step at 95 °C for 1 min and 30 s (1 cycle). This was followed by 40 cycles, each consisting of denaturation at 95 °C for 20 s, annealing at 64 °C for 20 s, and extension at 72 °C for 30 s. Finally, a melting curve analysis was performed, starting at 60 °C for 1 min and increasing by 0.4 °C every 15 s until 95 °C was reached, where it was maintained for 15 s (1 cycle). The primers employed in this study are presented in Table 1.

**Table 1:** Primer sequences.

Primer name	Type	Sequence (5′-3′)	Size (bp)
IL-6	F	CTGCAAGAGACTTCCATCCAG	155
	R	AGTGGTATAGACAGGTCTGTTGG	
TNF-α	F	CAGGCGGTGCCTATGTCTC	89
	R	CGATCACCCCGAAGTTCAGTAG	
Actin	F	CACCATTGGCAATGAGCGGTTC	130
	R	AGGTCTTTGCGGATGTCCACGT	

### 2.14 Western blot analysis

Cells were seeded in 6-well plates (4 × 10^5^ cells/well, 2 mL) and divided into the following groups: blank control, model, and XCHG-treated groups (low, medium, and high doses). After 24 h of culture, except for the control group, all groups were stimulated with LPS and treated with corresponding drug concentrations for 24 h Following treatment, cells were collected and total protein was extracted with RIPA buffer (30 min on ice), followed by centrifugation (12,000 rpm, 20 min, 4 °C) to obtain the supernatant. Samples were mixed with 5 × loading buffer and heat-denatured at 100 °C for 10 min. Equal protein quantities were separated by SDS-PAGE using 15-well precast gels and electrotransferred to PVDF membranes. Membranes were blocked for 30 min, then incubated overnight at 4 °C with primary antibodies against p-EGFR, EGFR, p-SRC, SRC, ESR1 (all at 1:1000 dilution), and GAPDH (1:5000). Following five TBST washes (6 min each), HRP-conjugated secondary antibodies (1:5000) were applied for 1 h at room temperature. After additional washing, bands were visualized by ECL and captured using a chemiluminescence imaging system. Band intensities were quantified with ImageJ.

### 2.15 Statistical analysis

The data are shown as mean ± standard deviation (SD). Statistical significance was assessed using one-way analysis of variance (ANOVA) via GraphPad Prism 10.1.2 software, with a *p*-value of less than 0.05 regarded as statistically significant.

## 3 Results

### 3.1 The blood components in XCHG have potential interactions with 120 shared targets of febrile diseases XCHG

**Figure 4:**
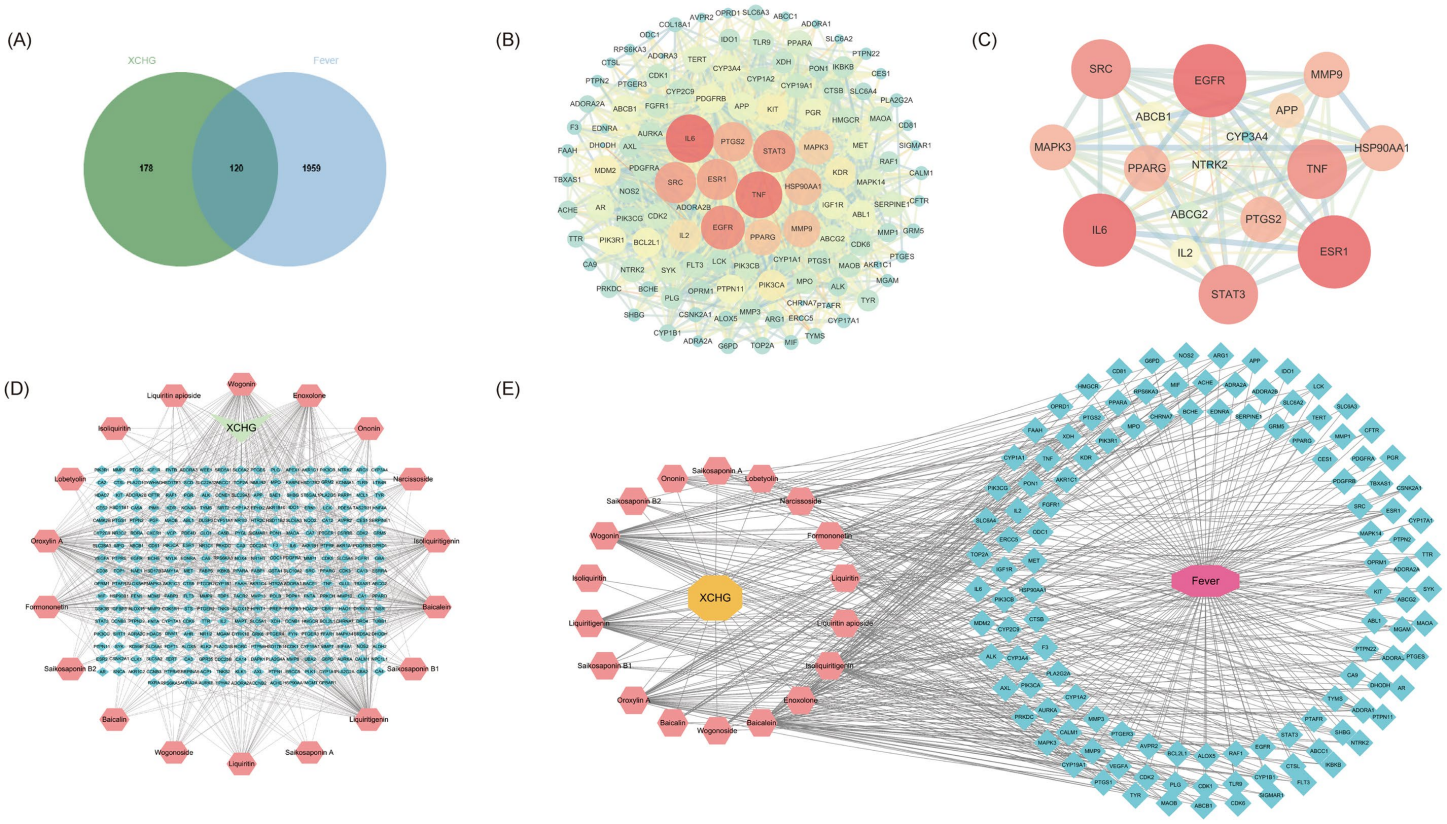
Multi-target network analysis. **(A)** Venn diagram of blood-absorbed components of XCHG and disease targets. **(B)** PPI network of 120 cross-targets. **(C)** Key target network. **(D)** Blood-absorbed component-target network of XCHG. **(E)** XCHG herb-components-targets-fever network diagram.

Potential therapeutic targets of XCHG were identified through database mining and comparative analysis. The SwissTargetPrediction platform yielded 298 putative targets for the 18 blood-absorbed components. Concurrently, fever-associated genes (*n* = 2,079) were compiled from four disease databases (GeneCards, OMIM, TTD, DrugBank) following deduplication. Intersection analysis through Venn plotting revealed 120 shared targets between component and disease gene sets (Figure 4A), representing potential fever-modulating targets of XCHG.

### 3.2 The PPI network identified 17 key targets among the above-mentioned shared

The 120 shared targets were analyzed via STRING to generate a PPI network (120 nodes, 1,432 edges; Figure 4B). Network visualization in Cytoscape 3.7.0 incorporated node attributes scaled by topological importance, with size and color intensity reflecting connectivity (Degree). Network centrality parameters—degree (DC), betweenness (BC), and closeness centrality (CC)—were computed via the CentiScape 2.2 plugin. Targets with DC, BC, and CC values exceeding their respective means (Degree ≥23.87, Closeness≥0.00446, Betweenness≥109.13) were defined as key targets, and a condensed PPI subnetwork was generated. Through this analysis, we identified 17 key targets (Figure 4C) including IL6, TNF(TNF-*α*), EGFR, STAT3, SRC, ESR1, PTGS2, HSP90AA1, MMP9, PPARG, MAPK3, IL2, CYP3A4, APP, ABCB1, ABCG2, and NTRK2. These targets demonstrated substantial network influence, implicating their functional significance in XCHG’s antipyretic activity. The top six key targets by Degree value (Table 2) were subsequently selected for molecular docking with key components.

**Table 2:** The top six key targets ranked by degree value.

Target name	Degree	Closeness centrality	Betweenness centrality
EGFR	16	1	0.02599597
IL6	16	1	0.02599597
ESR1	16	1	0.02599597
STAT3	15	0.94117647	0.01592653
SRC TNF-α	15 15	0.94117647 0.94117647	0.01592653 0.0209623

### 3.3 Topological network analysis identifies five core bioactive components mediating XCHG’S anti-fever effects

The “herb-component-target-fever” interaction network was constructed using Cytoscape 3.7.0 through topological analysis of overlapping targets. The blood-absorbed component-target network analysis revealed a network comprising 317 nodes, including 298 key targets, 18 components, and XCHG (Figure 4D), demonstrating the pharmacological relevance between bioavailable components and their molecular targets. The further constructed integrated “herb-component-target-fever” network comprised 140 nodes, including 120 key targets, 18 components, XCHG, and fever (Figure 4E). Based on degree values, key components were identified as primary therapeutic components: Oroxylin A, Wogonin, Baicalein, Liquiritigenin, and Enoxolone (Table 3). These components are predicted to be the major key components responsible for XCHG’s antipyretic effects.

**Table 3:** The top five components ranked by degree value.

Ingredient name	Degree	Closeness centrality	Betweenness centrality
Oroxylin A	105	0.43621399	0.13701609
Wogonin	105	0.43621399	0.15307644
Baicalein	105	0.43621399	0.17399126
Liquiritigenin	103	0.43383356	0.29743801
Enoxolone	93	0.42343542	0.33684036

### 3.4 Enrichment analysis supports the multi-target mechanism of XCHG

**Figure 5:**
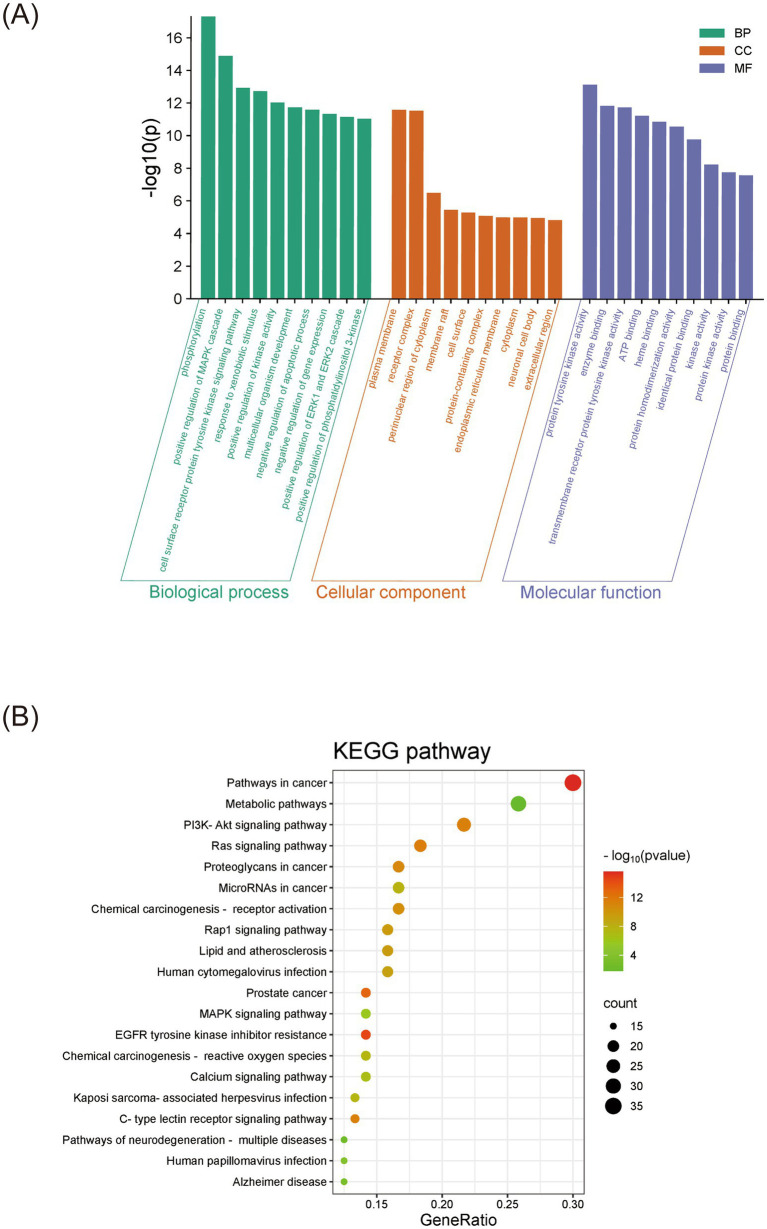
GO and KEGG enrichment analysis. **(A)** GO terms. **(B)** KEGG pathways.

Functional enrichment analysis revealed XCHG’s comprehensive regulatory profile across key biological domains. The analysis identified 528 significantly enriched biological processes (BP), prominently featuring protein phosphorylation and MAPK cascade regulation. Cellular component (CC) analysis (69 terms) demonstrated predominant localization to focal membrane receptor complexes and endoplasmic reticulum structures. Molecular function (MF) assessment (129 terms) highlighted crucial activities including tyrosine kinase function, ATP binding, and enzyme binding (Figure 5A). These findings collectively suggest that XCHG, while regulating cellular stress responses, also has the potential to regulate phosphorylation signaling (such as the MAPK cascade) and membrane receptor activity. KEGG pathway analysis from the DAVID database identified 160 pathways, with the top 20—including PI3K-Akt, Ras, Rap1, MAPK, C-type lectin receptor signaling, and EGFR tyrosine kinase inhibitor resistance (Figure 5B) —Although these pathways are broadly functional signaling cascades involved in diverse cellular processes, substantial evidence supports their specific roles in inflammatory fever pathophysiology. The PI3K-Akt pathway has been demonstrated to regulate macrophage inflammatory activation and cytokine production, with PI3K inhibition significantly attenuating LPS-induced TNF-*α* and IL-6 release (24). The Ras signaling pathway functions as an upstream activator of the MAPK cascade; upon Toll-like receptor (TLR) stimulation in macrophages, Ras activation triggers the Raf–MEK–ERK cascade, which promotes the transcription of pro-inflammatory cytokines including TNF-α and IL-6 through AP-1 activation (25, 26). The Rap1 signaling pathway, while primarily recognized for regulating integrin-mediated cell adhesion, has been shown to modulate inflammatory responses in immune cells by controlling leukocyte recruitment to inflammatory sites and regulating NF-κB-dependent cytokine production (27). The MAPK cascade, particularly p38 MAPK, directly controls COX-2 mRNA stability and protein expression in response to pyrogenic stimuli such as IL-1β (28). EGFR signaling has been shown to amplify inflammatory responses in macrophages through transactivation of NF-κB and subsequent upregulation of pro-inflammatory mediators (29). Furthermore, C-type lectin receptor pathways are pattern recognition receptors that detect pathogen-associated molecular patterns and initiate the inflammatory cascade leading to fever (30). Collectively, these pathways converge on the regulation of pro-inflammatory cytokine production (TNF-*α*, IL-1β, IL-6) and downstream mediators (COX-2, PGE2) that constitute the molecular basis of inflammatory fever. These findings suggest that XCHG may exert its antipyretic effects through modulation of these inflammation-related signaling networks, supporting its multi-target mechanism of action.

### 3.5 Molecular docking indicates high-affinity binding of key XCHG components to core fever targets

**Figure 6:**
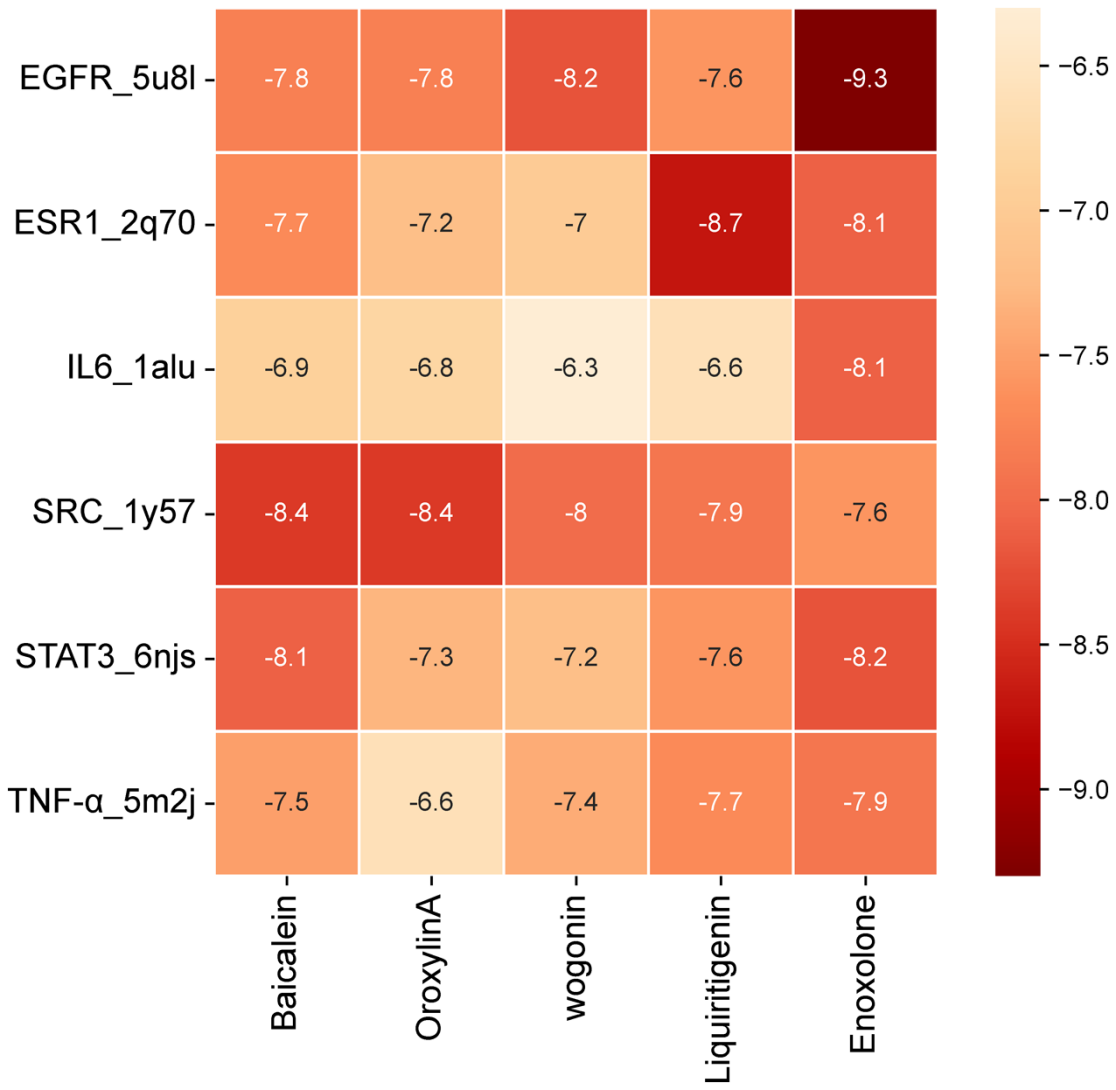
Binding energy (kcal/mol) heatmap of key components of XCHG and top six key target.

**Figure 7:**
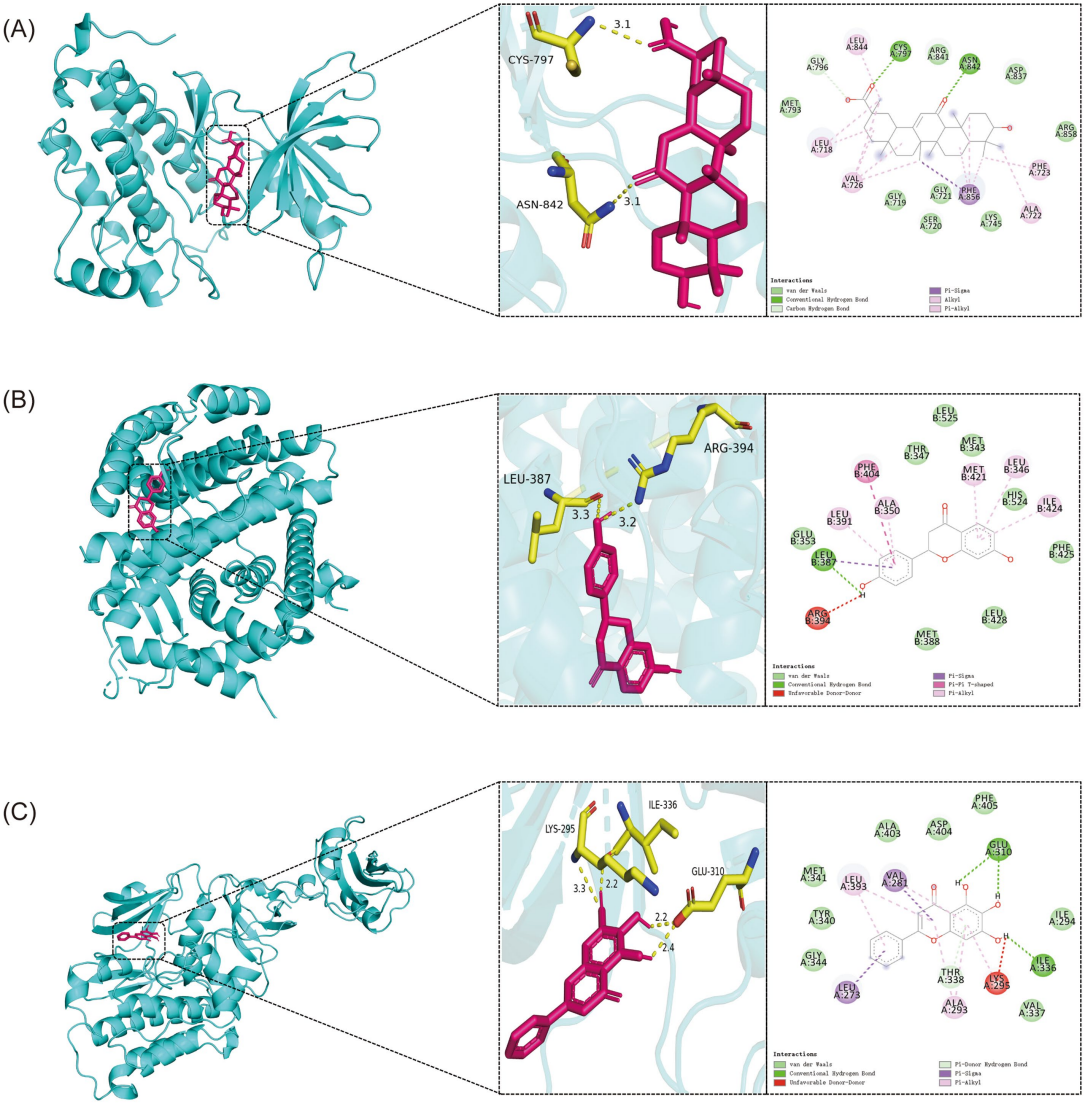
Molecular docking results and binding interaction analysis. The figure illustrates the three-dimensional binding conformations (protein shown in blue cartoon representation and ligand in stick model) and two-dimensional interaction patterns (green dashed lines for hydrogen bonds, light green circles for van der Waals interactions, and pink dashed lines for *π*-π stacking) of the protein-ligand complexes: **(A)** EGFR-Enoxolone, **(B)** ESR1-Liquiritigenin, **(C)** SRC-Baicalein.

Molecular docking between XCHG’s key components and six highest-degree targets (EGFR, IL6, ESR1, STAT3, SRC, TNF-α) revealed binding energies ranging from −6.3 to −9.3 kcal/mol (Figure 6), all exceeding the favorable binding threshold (−5.0 kcal/mol). Three complexes demonstrated exceptionally strong interactions: EGFR-Enoxolone (−9.3 kcal/mol), ESR1-Liquiritigenin (−8.7 kcal/mol), and SRC- Baicalein (−8.4 kcal/mol), with binding energies significantly below the −7.0 kcal/mol high-affinity threshold (31). Detailed interaction analysis revealed multi-modal binding patterns (Figure 7). The EGFR-Enoxolone complex showed hydrogen bonds with CYS797 and ASN842, extensive hydrophobic interactions with multiple residues, and *π*-*σ* interactions involving PHE856. ESR1-Liquiritigenin formed hydrogen bonds through LEU387, comprehensive hydrophobic contacts, and π-π stacking with PHE404. SRC-Baicalein exhibited hydrogen bonding via GLU310 and ILE336, hydrophobic interactions with key residues, and π-σ/π-alkyl interactions stabilizing the flavonoid structure. These multi-modal interaction patterns validate the strong binding capabilities of these compounds to their respective targets, explaining their exceptionally high binding affinities. These three optimal complexes were selected for further MD simulations to investigate their dynamic binding characteristics.

### 3.6 The complex of EGFR-Enoxolone, ESR1-Liquiritigenin and SRC-Baicalein can be stably formed

**Figure 8:**
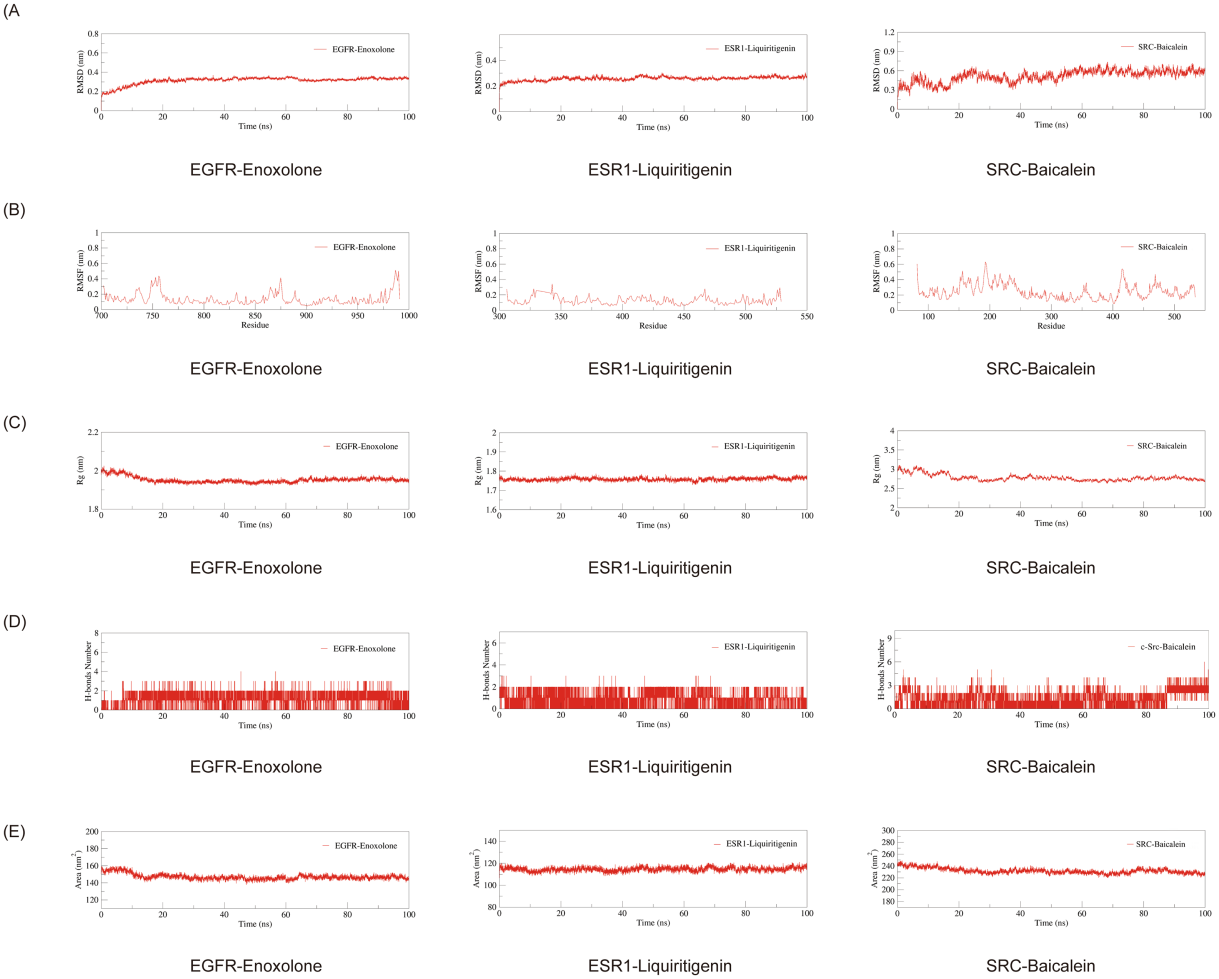
Molecular dynamics analysis of three protein-ligand complexes: **(A)** RMSD, **(B)** RMSF, **(C)** R_g_, **(D)** number of H-bonds, and **(E)** SASA.

The structural integrity of protein-ligand complexes was assessed through root-mean-square deviation (RMSD) analysis, with values under 1 nm signifying stable molecular interactions under physiological conditions (32). RMSD analysis demonstrated that all three complexes (EGFR-Enoxolone, ESR1-Liquiritigenin, and SRC-Baicalein) maintained high structural stability within 20-60 ns (Figure 8A), with RMSD values stabilizing at approximately 0.3 nm, 0.25 nm, and 0.4–0.6 nm, respectively. Compared to the other two complexes, SRC-Baicalein exhibited relatively larger RMSD fluctuations (0.4–0.6 nm), which may be attributed to inherent structural characteristics of different proteins under identical simulation conditions. RMSF was used to assess positional fluctuations of amino acid residues, reflecting regional flexibility (33). RMSF analysis revealed that all three complexes displayed overall fluctuations below1 nm, indicating limited residue mobility, with flexible regions exhibiting fluctuations between 0.4–0.6 nm (Figure 8B). Rg was utilized to evaluate overall structural compactness, where larger values indicate structural expansion while smaller values reflect tighter packing (34). Rg analysis further confirmed the structural compactness of the complexes, with Rg values maintained at approximately 2 nm, 1.75 nm, and 3 nm for the three complexes respectively, showing minimal fluctuations (Figure 8C). Hydrogen bonds (H-bonds), as critical non-covalent interactions governing complex stability (35), were quantitatively analyzed across the three complexes. The EGFR-Enoxolone complex formed 2 stable H-bonds, ESR1-Liquiritigenin exhibited 1–2 bonds, while SRC-Baicalein showed the most extensive network with 2–3 persistent H-bonds (Figure 8D). This H-bond patterning demonstrates stable ligand-receptor recognition, the SRC-Baicalein complex forming the most H-bonds, indicative of superior binding affinity. SASA reflects surface solvent accessibility, where stable SASA profiles indicate well-folded structures (36). All three complexes exhibited minimal SASA fluctuations (Figure 8E), further supporting their high structural stability. These results collectively demonstrate that all three complexes maintained excellent binding stability and structural compactness throughout the MD simulations.

### 3.7 The EGFR, ESR1 and SRC protein-ligand complexes demonstrated excellent conformational stability in molecular dynamics simulations

**Figure 9:**
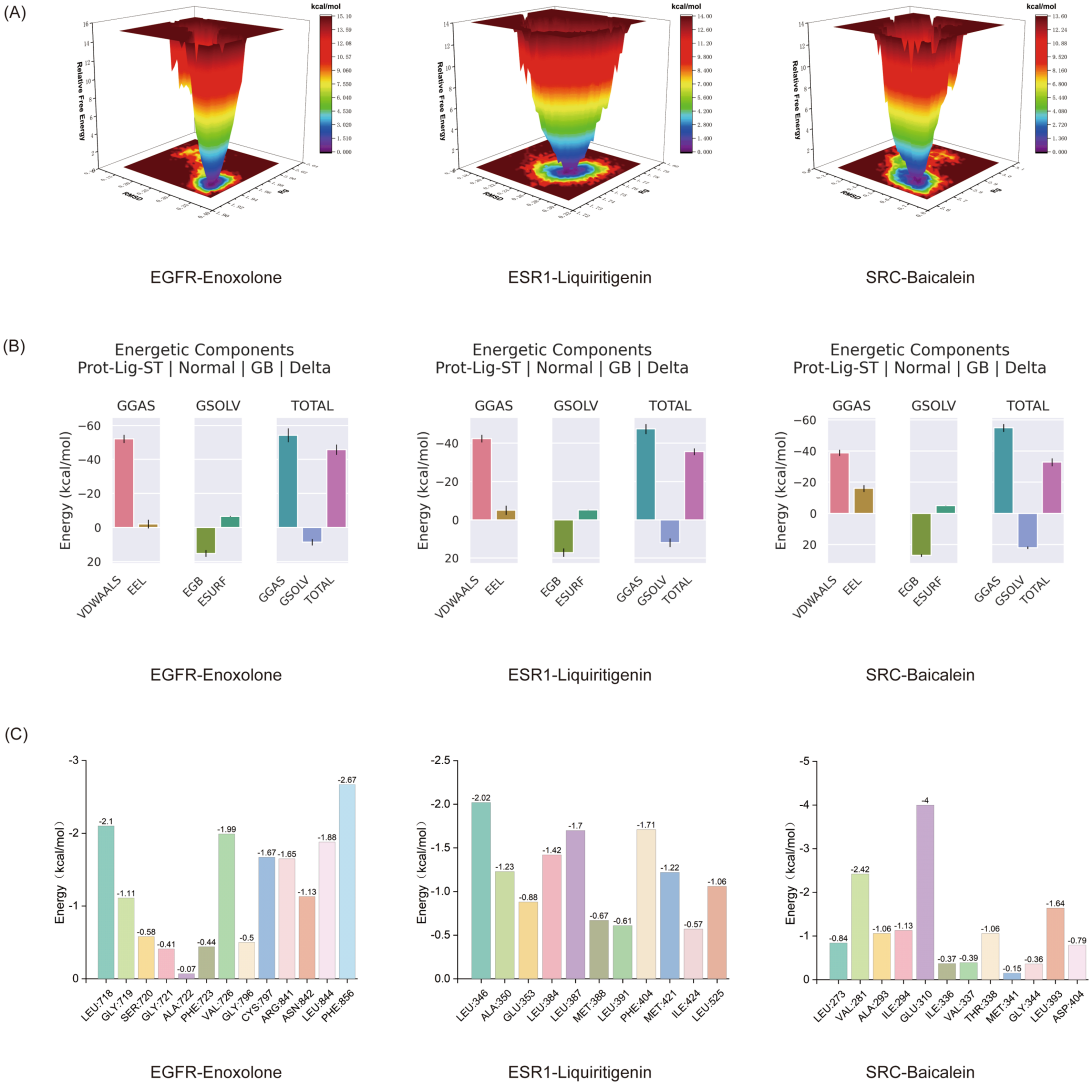
Comprehensive energetic analysis of three protein-ligand interactions. **(A)** FEL analysis. **(B)** The average binding free energy, along with its components—van der Waals interactions (VDWAALS), electrostatic energy (EEL), polar solvation energy (EGB), non-polar solvation energy (ESURF), molecular mechanics energy (GGAS), and solvation energy (GSOLV). **(C)** Residue energy contributions.

The FEL was generated using RMSD and Rg data to characterize conformational changes of the complexes during simulations. In the FEL plots, dark-colored regions represent the lowest-energy states while light-colored areas correspond to higher-energy states (37). The results demonstrated that all three complexes (EGFR-Enoxolone, ESR1-Liquiritigenin, and SRC-Baicalein) formed single, well-defined low-energy clusters (Figure 9A), indicating high conformational stability and strong binding interactions during the simulations. The MM/PBSA approach quantified binding affinities, with more negative values corresponding to stronger receptor-ligand interactions (38). The MM/PBSA calculations revealed strongly favorable binding free energies for all three complexes (Figure 9B; Supplementary Table S4): EGFR-Enoxolone (−45.79 kcal/mol), ESR1-Liquiritigenin (−35.58 kcal/mol), and SRC-Baicalein (−32.9 kcal/mol), indicating robust molecular recognition at the binding interfaces. Per-residue energy decomposition analysis (Figure 9C) identified a key binding site residue that made a significant contribution to the interaction: PHE856 (−2.67 kcal/mol), LEU718 (−2.1 kcal/mol), and VAL726 (−1.99 kcal/mol) in EGFR-Enoxolone; LEU346 (−2.02 kcal/mol), PHE404 (−1.71 kcal/mol), and LEU387 (−1.7 kcal/mol) in ESR1-Liquiritigenin; and GLU310 (−4.0 kcal/mol) in SRC-Baicalein. The smaller the values corresponding to these residues, the greater their contribution to the binding energy. These energetically favorable interactions significantly stabilized the respective complexes. All three systems demonstrated excellent conformational and binding stability throughout molecular dynamics simulations, providing crucial insights for future studies of protein-ligand interactions.

### 3.8 Dynamic signatures of XCHG complexes: dominant motions (PCA), residue correlations (DCCM), and stable binding explain SRC-Baicalein’s superiority

**Figure 10:**
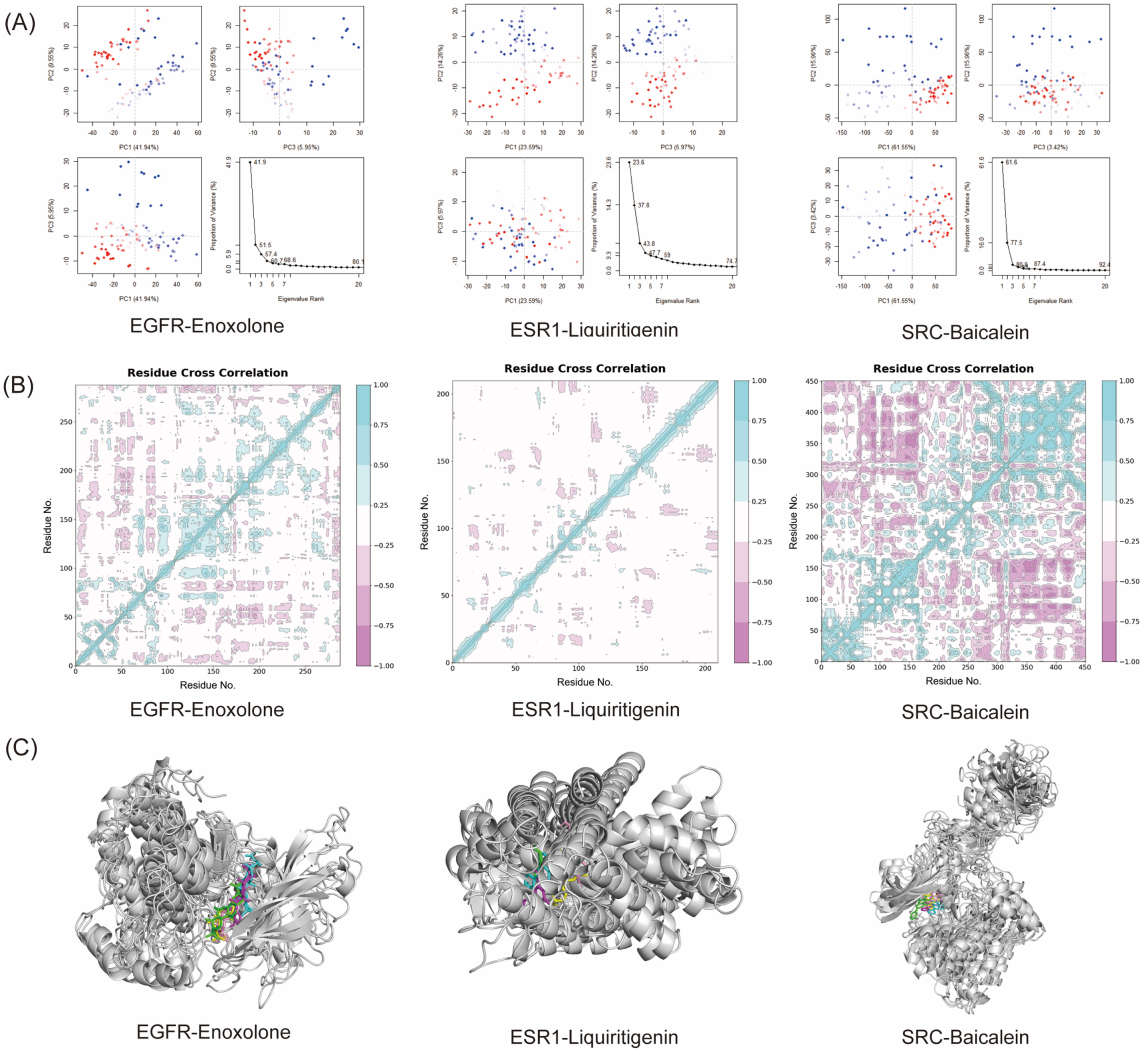
Conformational and structural analysis of three protein-ligand complexes during molecular dynamics simulation. **(A)** PCA analysis. **(B)** DCCM analysis. **(C)** Structural analysis at 0 s, 25 ns, 50 ns, 75 ns, and 100 ns during the molecular dynamics simulation.

To further investigate the conformational changes of proteins in the complexes, we employed PCA and DCCM to evaluate the dynamic behavior of three receptor-ligand systems. PCA identified dominant motion modes, with plot axes representing conformational space dimensions and color gradients (blue to red) indicating simulation progression time (39). DCCM revealed residue-residue cross-correlations, where color gradients (light blue to pink) represented motion coordination: values approaching 1 indicated synchronized movements, while values near −1 represented anti-correlated motions (40, 41) PCA results (Figure 10A) demonstrated that the three principal components (PC1, PC2, and PC3) accounted for 57.44, 43.82, and 80.93% of the total variance in the EGFR-Enoxolone, ESR1-Liquiritigenin, and SRC-Baicalein complexes, respectively. PC1 primarily captured large-scale conformational changes, while PC2 and PC3 were associated with medium-range motions and localized movements, respectively. Notably, the SRC-Baicalein complex exhibited the highest PC1 contribution (61.55%), indicating its most pronounced conformational flexibility and suggesting greater potential for dynamic responses and structural adaptability. DCCM analysis (Figure 10B) revealed distinct binding dynamics among the complexes. In the EGFR-Enoxolone complex, correlated motions were predominantly observed within residues 100–150 and 200–250, demonstrating significant cooperative movements in these regions with relatively independent motions elsewhere. The ESR1-Liquiritigenin complex showed strongly coupled dynamics confined to residues 50–100, while other regions displayed more independent motions. Notably, the SRC-Baicalein complex demonstrated the most pronounced correlation patterns, with strong positive couplings (residues 100–150) and anti-correlated motions (residues 300–450) that significantly exceeded those observed in other complexes. This unique dynamic profile, characterized by enhanced residue coordination and pronounced anti-correlations, likely underpins both its superior binding affinity and exceptional conformational adaptability during simulations. Furthermore, structural snapshots extracted at 0, 25, 50, 75, and 100 ns time points from the molecular dynamics trajectories (Figure 10C) demonstrated EGFR-Enoxolone, ESR1-Liquiritigenin, and SRC-Baicalein maintained stable binding conformations without significant positional drift, confirming their excellent binding stability. Collectively, these findings provide crucial insights into the dynamic behavior and binding characteristics of the protein-ligand complexes, while supporting the therapeutic potential of these ligands as drug candidates.

### 3.9 mg/mL is used as a safety threshold to guide the concentration selection for cytotoxic experiments

The selection of RAW264.7 macrophages as the *in vitro* model was based on several considerations. First, macrophages serve as sentinel cells in the innate immune system and are primary producers of pyrogenic cytokines (TNF-*α*, IL-1β, IL-6) upon pathogen recognition, directly initiating the fever cascade (42). Second, KEGG pathway enrichment analysis revealed significant involvement of PI3K-Akt, MAPK, and EGFR signaling pathways, all of which have been extensively documented to regulate macrophage inflammatory activation and cytokine secretion (26). Third, the enrichment of C-type lectin receptor signaling pathways further supports the relevance of macrophage models, as these pattern recognition receptors are predominantly expressed on macrophages and mediate pathogen-induced inflammatory responses (30). Fourth, RAW264.7 cells represent a well-established and widely validated model for studying anti-inflammatory mechanisms of natural products, enabling comparison with previous studies on herbal medicine compounds (43). Therefore, this cellular model provides a biologically relevant platform to investigate XCHG’s modulatory effects on the inflammatory-pyrogenic signaling network.

**Figure 11:**
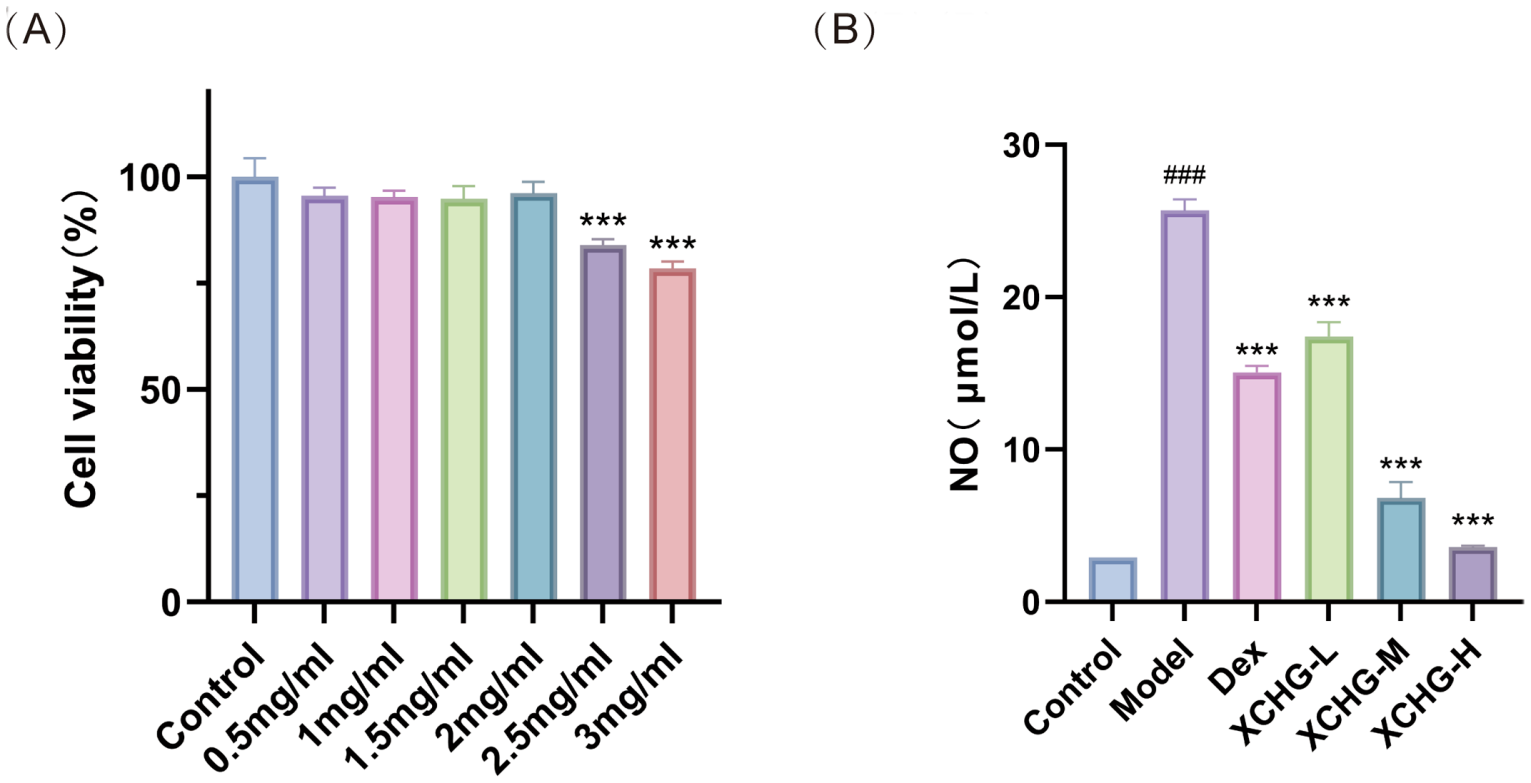
Cytotoxicity and NO inhibitory effects of XCHG on RAW 264.7 macrophages. **(A)** Cell viability after 24 h treatment with XCHG (0–3 mg/mL) determined by CCK-8 assay. **(B)** NO production in LPS-stimulated cells (1 μg/mL) treated with varying XCHG concentrations (low, medium, high) for 24 h. Data are presented as mean ± SD (*n* = 3). ^###^*p* < 0.001 vs. control, ^***^ *p* < 0.001 vs. model.

The CCK-8 assay results (Figure 11A) demonstrated that XCHG at concentrations below 2 mg/mL had minimal impact on RAW264.7 cell viability after 24-h treatment compared to the control group. However, cytotoxicity of the drug has been observed when the concentration exceeds 2 mg/mL. Therefore, we do not include this dose range to avoid interference caused by the cytotoxic mechanism. Based on these findings, subsequent experiments were conducted using XCHG concentrations of 1, 1.5, and 2 mg/mL.

### 3.10 XCHG dose-dependently inhibits LPS-induced NO production in RAW264.7 cells

NO is usually used to characterize the activation of inflammatory responses in RAW264.7 macrophages. As determined by Griess assay, XCHG significantly attenuated LPS-induced NO production in RAW264.7 cells (Figure 11B). The LPS-induced model group showed markedly increased NO secretion compared to the control group (*p* < 0.001), confirming successful model establishment. Notably, XCHG treatment at all tested concentrations (low dose: 1 mg/mL, medium dose: 1.5 mg/mL, and high dose: 2 mg/mL) and Dex dose-dependently suppressed NO release compared to the model group, demonstrating potent anti-inflammatory effects.

### 3.11 XCHG concentration-dependent inhibits the expression of key pro-inflammatory factors in LPS-induced RAW264.7 cells

**Figure 12:**
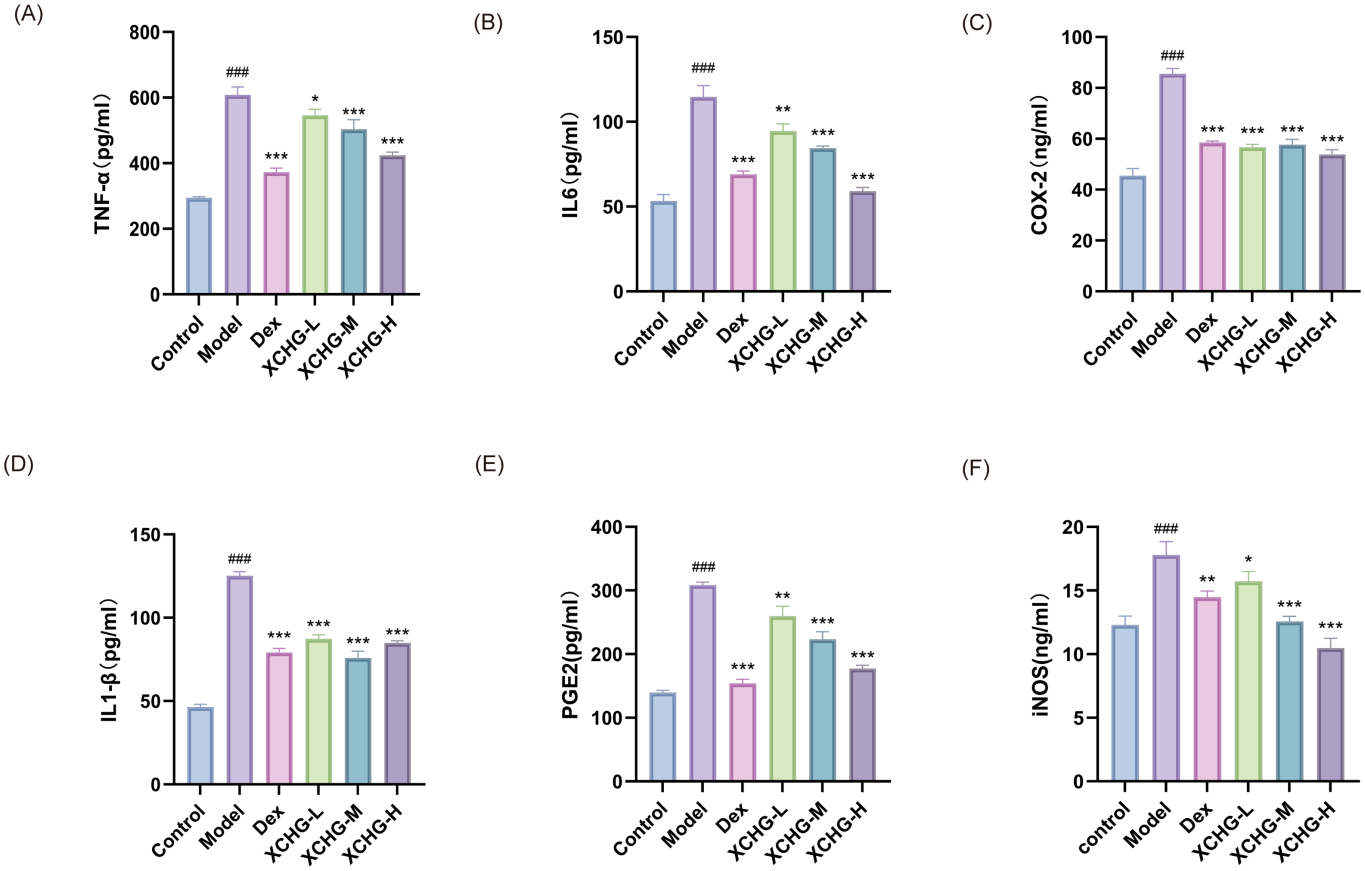
Effects of XCHG on inflammatory mediators in LPS-induced RAW264.7 cells. The levels of inflammatory mediators in culture supernatants were measured by ELISA. **(A)** TNF-*α*. **(B)** IL-6. **(C)** COX-2. **(D)** IL-1β. **(E)** PGE2. **(F)** iNOS. Data are presented as mean ± SD (*n* = 3). ^###^*p* < 0.001 vs. control, ^*^ *p <* 0.05 vs. model, ^**^ *p <* 0.01 vs. model. ^***^ *p <* 0.001 vs. model.

Figure 12 demonstrates that the LPS-treated model group exhibited significantly elevated levels of inflammatory biomarkers—TNF-*α*, IL-6, COX-2, IL-1β, PGE2, and iNOS—compared to untreated controls (*p* < 0.001). Both Dex and medium-to-high doses of XCHG significantly suppressed the expression of TNF-α and iNOS (*p* < 0.001), while the low-dose XCHG group showed moderate reduction in these parameters (*p* < 0.05). Moreover, treatment with Dex or any concentration of XCHG resulted in substantial suppression of IL-6, COX-2, IL-1β, and PGE2 relative to the LPS-induced group. These findings reveal that XCHG inhibits the release of pro-inflammatory factors induced by LPS in macrophages in a dose-dependent manner, thereby suppressing their inflammatory activation. Notably, the immunomodulatory efficacy of medium- and high-dose XCHG treatment approached that of the clinical reference compound Dex.

### 3.12 Suppression of LPS-induced TNF-α and IL-6 mRNA by XCHG treatment

**Figure 13:**
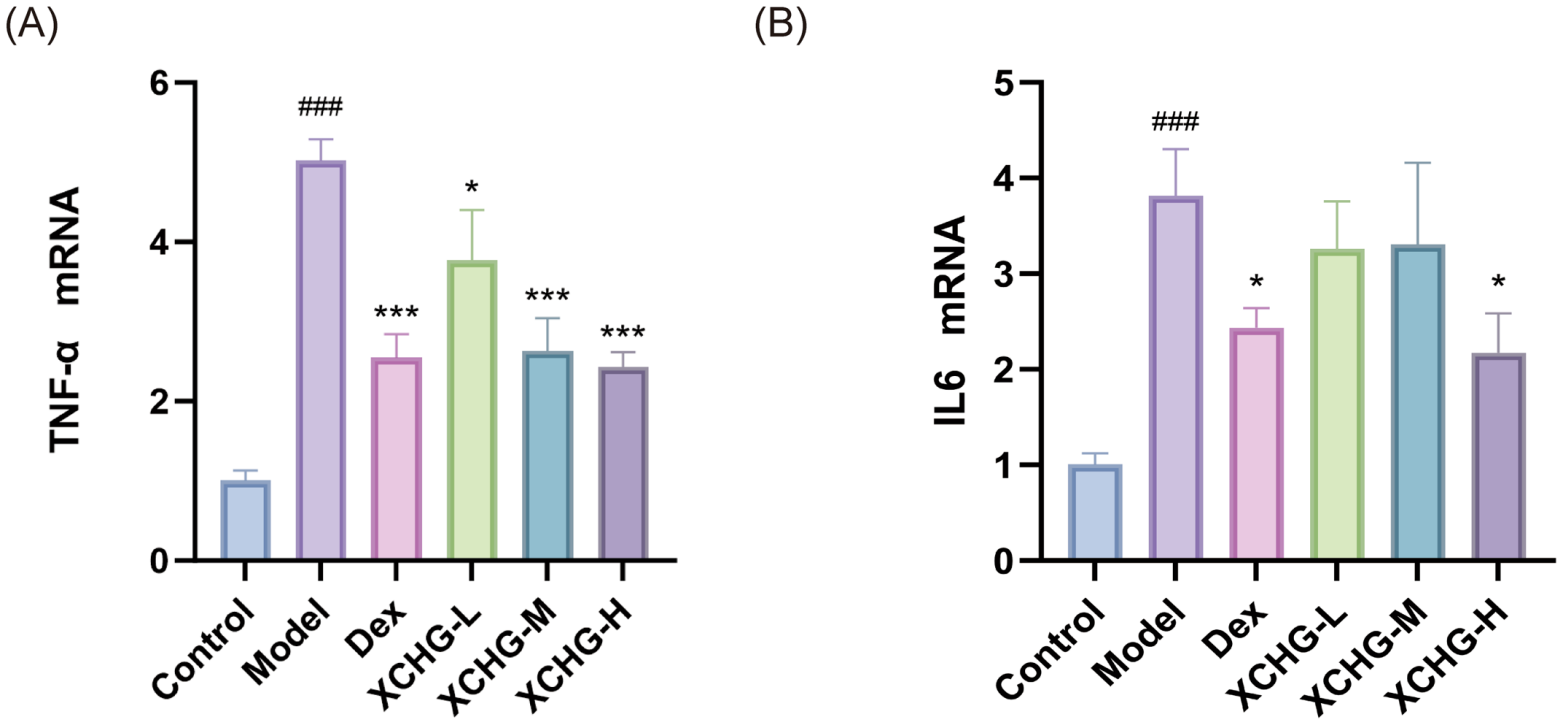
Effect of XCHG on TNF-α and IL-6 mRNA levels in LPS-induced RAW264.7 cells. Total RNA was extracted and mRNA expression levels were analyzed by quantitative real-time PCR (qRT-PCR). **(A)** Effect of TNF-α mRNA expression. **(B)** Effect of IL-6 mRNA expression. Data are presented as mean ± SD (*n* = 3). ^###^*p* < 0.001 vs. control, ^*^ *p <* 0.05 vs. model, ^***^ *p <* 0.001 vs. model.

As shown in Figure 13, compared with the control group, the LPS-induced model group exhibited significantly upregulated mRNA expression levels of TNF-α and IL-6 (*p* < 0.001). Relative to the LPS model group, both the Dex group and the medium-and high-dose XCHG groups demonstrated marked downregulation of TNF-α mRNA expression (*p* < 0.001). Additionally, the Dex group and high-dose XCHG group significantly suppressed IL-6 mRNA levels (*p* < 0.05).

### 3.13 Western blot analysis confirms XCHG modulates EGFR, SRC, and ESR1 at the protein level

**Figure 14:**
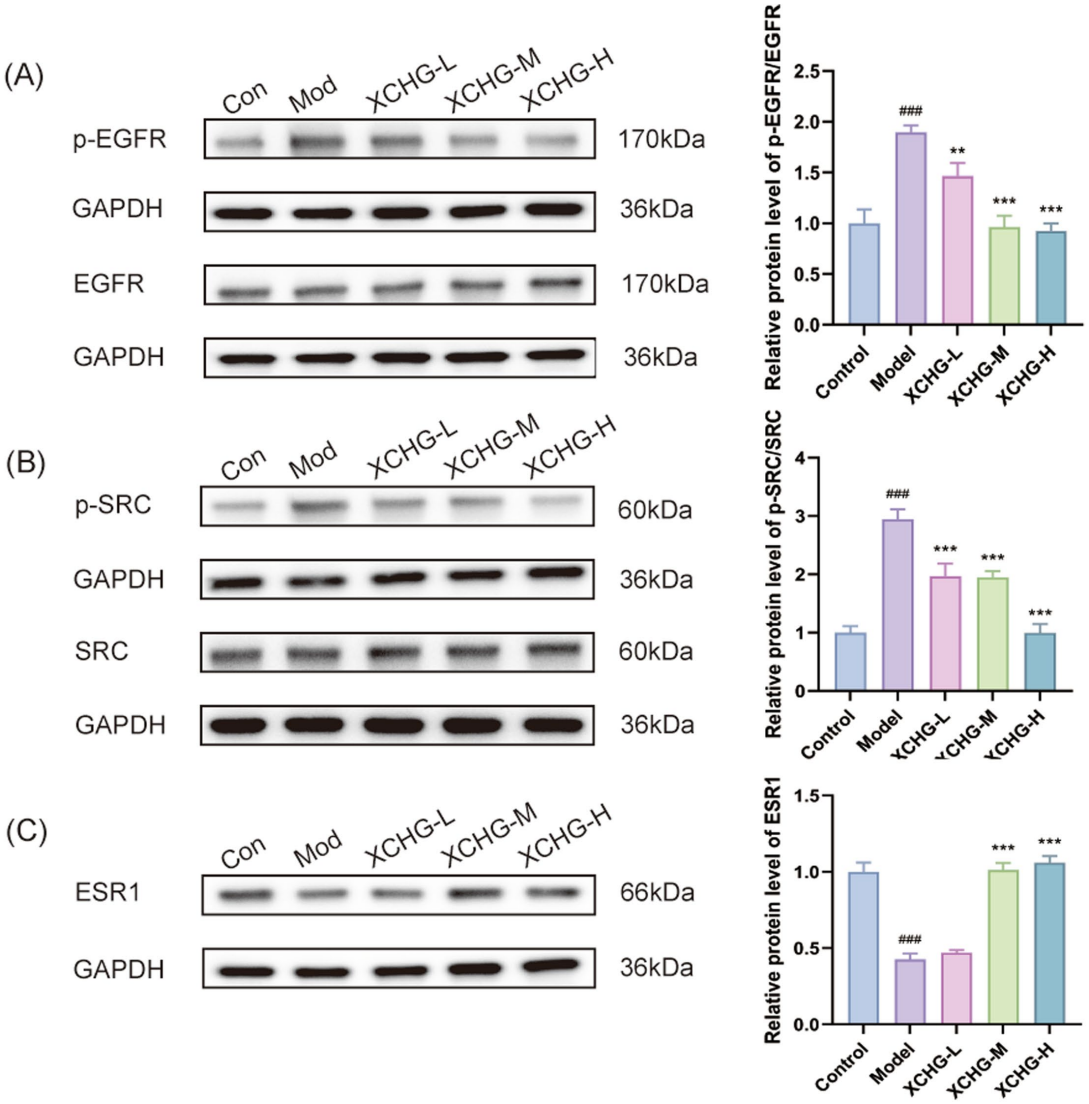
Effects of XCHG’s effects on predicted core targets in LPS-induced RAW264.7 macrophages. **(A)** P-EGFR/EGFR ratio. **(B)** P-SRC/SRC ratio, and **(C)** ESR1 expression. Data are presented as mean ± SD (*n* = 3). ^###^*p* < 0.001 vs. control, ^**^p < 0.01 vs. model, ^***^p < 0.001 vs. model.

Western blot analysis revealed that (Figure 14), compared with the control group, the model group exhibited significantly elevated p-EGFR/EGFR and p-SRC/SRC ratios, accompanied by a marked reduction in ESR1 expression (all *p* < 0.001). XCHG treatment attenuated the p-EGFR/EGFR ratio in a dose-dependent manner, with the low-dose group demonstrating moderate inhibition (*p* < 0.01), while the medium- and high-dose groups exhibited more pronounced suppression (*p* < 0.001) (Figure 14A). Regarding SRC phosphorylation, all three doses of XCHG significantly suppressed the p-SRC/SRC ratio (*p* < 0.001) (Figure 14B), indicating a sustained inhibitory effect on SRC activation across the dosage range. Furthermore, both medium- and high-dose XCHG treatments significantly restored ESR1 expression (*p* < 0.001) (Figure 14C), suggesting a protective effect of XCHG on ESR1 protein levels. Collectively, these findings demonstrate that XCHG intervention effectively suppresses aberrant activation of the EGFR-SRC signaling pathway while restoring ESR1 expression in model cells.

## 4 Discussion

Blood-absorbed components constitute the material basis for a drug’s pharmacological effects, as only compounds entering systemic circulation can interact with biological targets to exert therapeutic actions (44, 45). Our previous pharmacokinetic study identified 18 components of XCHG that enter systemic circulation in rats, predominantly flavonoids and triterpenoid saponins with documented roles in immune modulation and inflammatory regulation. Specifically, baicalin suppresses the NLRP3 inflammasome (46), baicalein inhibits COX-2 and iNOS expression (47), and lobetyolin demonstrates immunomodulatory effects through glutamine metabolism regulation (48). These findings suggested that XCHG’s therapeutic effects on inflammation and pyrexia likely arise from synergistic actions of multiple components targeting diverse pathways.

A critical innovation of this study lies in focusing exclusively on blood-absorbed components rather than all herbal constituents—a fundamental requirement often overlooked in traditional Chinese medicine research. Previous network pharmacology studies typically included all reported phytochemicals regardless of bioavailability, potentially introducing false-positive predictions (49). Our approach ensures that only compounds with demonstrated systemic exposure are analyzed, aligning with the established principle that only circulating compounds can interact with biological targets. Building on these components, network pharmacology revealed 120 shared targets between XCHG and fever-associated genes, PPI analysis refined these to 17 core targets, and topological analysis identified five key bioactive compounds: Oroxylin A, Wogonin, Baicalein, Liquiritigenin, and Enoxolone. GO and KEGG enrichment analyses demonstrated significant associations with inflammatory processes and key signaling pathways, including PI3K-Akt, MAPK, and C-type lectin receptor signaling. This methodological rigor distinguishes our work from studies documenting individual component activities in isolation, revealing instead how these components synergistically modulate overlapping inflammatory pathways.

The identification of EGFR, SRC, and ESR1 as core targets represents a conceptual advance in understanding XCHG’s antipyretic mechanism. While previous fever research focused predominantly on classical pyrogenic pathways (IL-1β/IL-6/COX-2/PGE2) (50), our study reveals that XCHG operates by targeting upstream regulatory nodes that orchestrate these cascades. To validate this hypothesis computationally, molecular docking between the five key components and six high-degree targets identified three component-target pairs with exceptional binding affinity: EGFR-Enoxolone (−9.3 kcal/mol), ESR1-Liquiritigenin (−8.7 kcal/mol), and SRC-Baicalein (−8.4 kcal/mol). While molecular docking has become standard in natural product research, we extended this with 100-ns molecular dynamics simulations to provide critical dynamic validation often absent in traditional Chinese medicine studies. Previous computational studies relied solely on docking scores, which capture only initial binding poses without accounting for conformational dynamics or binding kinetics (51). Our comprehensive MD analyses—including RMSD stability, RMSF flexibility profiling, hydrogen bond persistence, MM/PBSA free energy calculations, and PCA/DCCM characterization—confirmed that all three complexes maintained high structural stability throughout physiological timescales, with binding free energies of −45.79, −35.58, and −32.9 kcal/mol, respectively. Notably, PCA revealed that the SRC-Baicalein complex exhibits the highest conformational variance (PC1 = 61.55%), indicating structural adaptability while maintaining binding stability. This dynamic flexibility, coupled with the most extensive hydrogen bonding network (2–3 persistent bonds), suggests that Baicalein achieves optimal binding through induced-fit mechanisms rather than rigid lock-and-key interactions.

Having established the computational foundation, we next sought to validate the functional consequences of XCHG treatment at the cellular level. In LPS-induced RAW264.7 macrophages, XCHG dose-dependently inhibited NO production and reduced levels of TNF-*α*, IL-6, COX-2, IL-1β, PGE2, and iNOS, with medium and high doses achieving efficacy comparable to dexamethasone; qPCR further corroborated substantial downregulation of IL-6 and TNF-α transcription. These results demonstrate that XCHG exerts potent anti-inflammatory effects, but the underlying molecular mechanism linking these functional outcomes to the predicted targets remained to be elucidated.

To address this critical question, we performed Western blot analysis to directly examine whether XCHG modulates the computationally predicted core targets. The results provided compelling experimental validation: XCHG treatment dose-dependently suppressed p-EGFR and p-SRC levels while total EGFR and SRC protein levels remained unchanged, indicating specific inhibition of kinase activation rather than protein degradation. Furthermore, ESR1 expression was significantly upregulated in a dose-dependent manner. These findings establish EGFR, SRC, and ESR1 as bona fide molecular targets of XCHG’s anti-inflammatory action, thereby bridging the computational predictions with the observed functional outcomes.

The mechanistic implications of these findings are significant. EGFR-SRC signaling is a critical driver of inflammatory amplification, propagating signals through transactivation of ERK1/2, PI3Kδ/Akt, and NF-κB (52, 53). The demonstrated inhibition of this axis represents a fundamental mechanistic distinction from conventional NSAIDs, which primarily inhibit downstream COX enzymes allowing upstream inflammatory signals to persist (54). Through convergent modulation of upstream nodes, XCHG may achieve broader immunomodulatory effects while potentially avoiding the gastrointestinal toxicity associated with COX-1 inhibition. Additionally, ESR1 upregulation provides molecular evidence for balanced inflammatory responses, as ESR1 regulates innate immunity by controlling the equilibrium between pro- and anti-inflammatory cytokine production (55). Unlike agents that simply suppress inflammation through receptor blockade, XCHG enhances ESR1 expression, potentially promoting active immune homeostasis—a mechanism distinct from corticosteroids, which broadly suppress immunity.

Integrating all our findings within the context of fever pathophysiology provides a comprehensive mechanistic picture. The pathophysiology of fever involves cascading reactions between inflammatory factors (56): macrophages release TNF-*α* and IL-1β upon pathogen encounter, inducing IL-6 production (57, 58); IL-1β activates COX-2 through NF-κB nuclear translocation and stabilizes COX-2 mRNA via p38 MAPK (59); IL-6 acts on the hypothalamic thermoregulatory center (1); COX-2 catalyzes PGE2 synthesis (60), elevating the thermostatic set point (61); and iNOS-derived NO upregulates COX-2 (62). Our results demonstrate that XCHG intervenes at the apex of this cascade through inhibition of p-EGFR and p-SRC, preemptively attenuating the entire inflammatory-pyrogenic signaling network; concurrently, ESR1 upregulation promotes inflammation resolution and immune homeostasis restoration by regulating the balance between pro- and anti-inflammatory cytokines, thereby achieving multi-layered, comprehensive intervention across the inflammatory-pyrogenic axis—from upstream signal blockade to downstream mediator suppression to immune balance regulation.

Building upon this mechanistic framework, it is important to acknowledge that the KEGG pathways identified in this study, including PI3K-Akt, MAPK, Ras, Rap1, C-type lectin receptor and EGFR signaling, are broadly functional cascades involved in diverse cellular processes beyond inflammation. However, their specific roles in the pyrogenic cascade have been well-documented. The PI3K-Akt pathway modulates NF-κB-dependent transcription of TNF-α and IL-6 in activated macrophages (63). The Ras cascade transduces LPS signals through the Ras/Raf-1/MEK/ERK pathway to activate TNF-α transcription (25, 26). Rap1 GTPase regulates macrophage integrin activation and phagocytosis in response to inflammatory stimuli (64). C-type lectin receptors activate Syk-CARD9-NF-κB signaling to induce pyrogenic cytokines (65). EGFR transactivation amplifies TLR4-mediated inflammatory signaling during bacterial infections (29). Therefore, their convergent regulation of the cytokine-COX-2-PGE2 axis provides a mechanistic rationale for XCHG’s antipyretic action.

Our KEGG analysis substantiates this multi-pathway mechanism, revealing enrichment in PI3K-Akt, MAPK, Ras, Rap1, EGFR, and C-type lectin receptor pathways, all converging on inflammatory gene transcription through NF-κB and AP-1 activation (66, 67). The demonstrated inhibition of p-EGFR provides direct mechanistic support for PI3K-Akt suppression, as EGFR-PI3K-Akt signaling is a key driver of inflammatory macrophage activation (29, 68). Importantly, preservation of macrophage viability at therapeutic doses indicates selective pathway modulation rather than cytotoxicity—crucial for maintaining antimicrobial defenses while dampening excessive inflammation. The reduction in p-SRC substantiates effects on the MAPK cascade, with COX-2 reduction particularly implicating p38 MAPK inhibition (59). The enrichment of C-type lectin receptor pathways potentially explains XCHG’s efficacy across diverse fever etiologies including viral infections (69).

This mechanistic profile contrasts favorably with conventional antipyretics: NSAIDs target only COX-2 with gastrointestinal risks (6), while corticosteroids carry immunosuppression risks (70). XCHG’s multi-target approach may achieve comparable efficacy with better safety profiles, as supported by favorable adverse event profiles in clinical trials (10, 11). The identification of five key components offers targets for quality control standardization, ensuring batch consistency and predicting clinical effectiveness. Beyond XCHG itself, our integrated framework—prioritizing blood-absorbed components and combining network pharmacology with molecular dynamics validation and multi-level experimental verification—establishes a replicable paradigm for elucidating mechanisms of traditional Chinese medicine formulations, addressing the disconnect between computational predictions and biological reality and providing rigorous evidence for regulatory approval in evidence-based medicine frameworks.

Despite our comprehensive methodology, several limitations warrant consideration. First, while network pharmacology analysis identified five key bioactive compounds (Oroxylin A, Wogonin, Baicalein, Liquiritigenin, and Enoxolone) and predicted their interactions with core targets, the experimental validation was performed using the whole XCHG formula rather than individual compounds. Therefore, the network pharmacology predictions should be interpreted as hypothesis-generating rather than conclusively established mechanisms. The specific contributions of individual compounds to the observed effects, and whether these compounds act synergistically or independently, remain to be determined through future studies using isolated compounds or targeted knockdown/knockout approaches. Second, animal fever models with continuous temperature monitoring are needed to validate *in vivo* efficacy and central thermoregulatory effects. Third, although Western blot analysis confirmed that XCHG modulates EGFR, SRC, and ESR1 at the protein level, direct evidence linking specific predicted compounds to these target modulations is lacking. Future studies employing surface plasmon resonance (SPR), cellular thermal shift assay (CETSA), or genetic manipulation approaches would strengthen the causal relationship between predicted compound-target interactions and observed functional outcomes. Finally, validation across diverse cell types would more comprehensively characterize XCHG’s anti-inflammatory profile. We are committed to addressing these limitations in future studies to further refine our understanding of XCHG’s anti-inflammatory and antipyretic mechanisms.

## 5 Conclusion

This study investigated the antipyretic mechanism of XCHG through an integrated computational-experimental approach. Using network pharmacology based on 18 pharmacokinetically validated blood-absorbed components, we identified five bioactive compounds (Oroxylin A, Wogonin, Baicalein, Liquiritigenin, and Enoxolone) that are predicted to target core inflammatory regulators (EGFR, ESR1, SRC) with high binding affinity and stability, as suggested by molecular docking and 100-ns molecular dynamics simulations. KEGG enrichment analysis identified several significantly enriched pathways, including PI3K-Akt, MAPK, Ras, Rap1, C-type lectin receptor, and EGFR signaling. Although these are broadly functional signaling cascades, they have been well-documented to play critical roles in regulating inflammatory responses and pyrogenic cytokine production. Western blot analysis provided direct experimental validation, demonstrating that XCHG dose-dependently suppresses p-EGFR and p-SRC phosphorylation while upregulating ESR1 expression. Functional validation in LPS-stimulated RAW264.7 macrophages further confirmed that XCHG potently inhibits inflammatory mediators (TNF-*α*, IL-1β, IL-6, COX-2, iNOS, PGE2, NO). Based on our findings, we propose that XCHG exerts comprehensive intervention across the inflammatory-pyrogenic axis through a dual mechanism: inhibition of p-EGFR and p-SRC blocks upstream inflammatory amplification, while ESR1 upregulation promotes immune homeostasis restoration—potentially distinguishing XCHG from conventional single-target antipyretics that only suppress downstream COX enzymes. However, it should be noted that while the whole formula demonstrated clear modulation of these targets, the specific contributions of individual predicted compounds to these effects require further experimental validation.

## Data Availability

The original contributions presented in the study are included in the article/Supplementary material, further inquiries can be directed to the corresponding authors.

## Footnotes

1
https://pubchem.ncbi.nlm.nih.gov/

2
http://www.swisstargetprediction.ch/

3
https://www.omim.org/

4
http://db.idrblab.net/ttd/

5
https://go.drugbank.com/

6
https://www.genecards.org/

7
https://www.bioinformatics.com.cn/

8
https://www.string-db.org/

9
https://david.ncifcrf.gov/
