# Supplementary figures and images for: Integrating network pharmacology, molecular docking and dynamics simulation to decipher the antipyretic mechanisms of Xiaochaihu granules
Source: Front Med (Lausanne). 2026 Feb 20;13:1772991. doi: 10.3389/fmed.2026.1772991 (PMC12962956; doi:10.3389/fmed.2026.1772991)

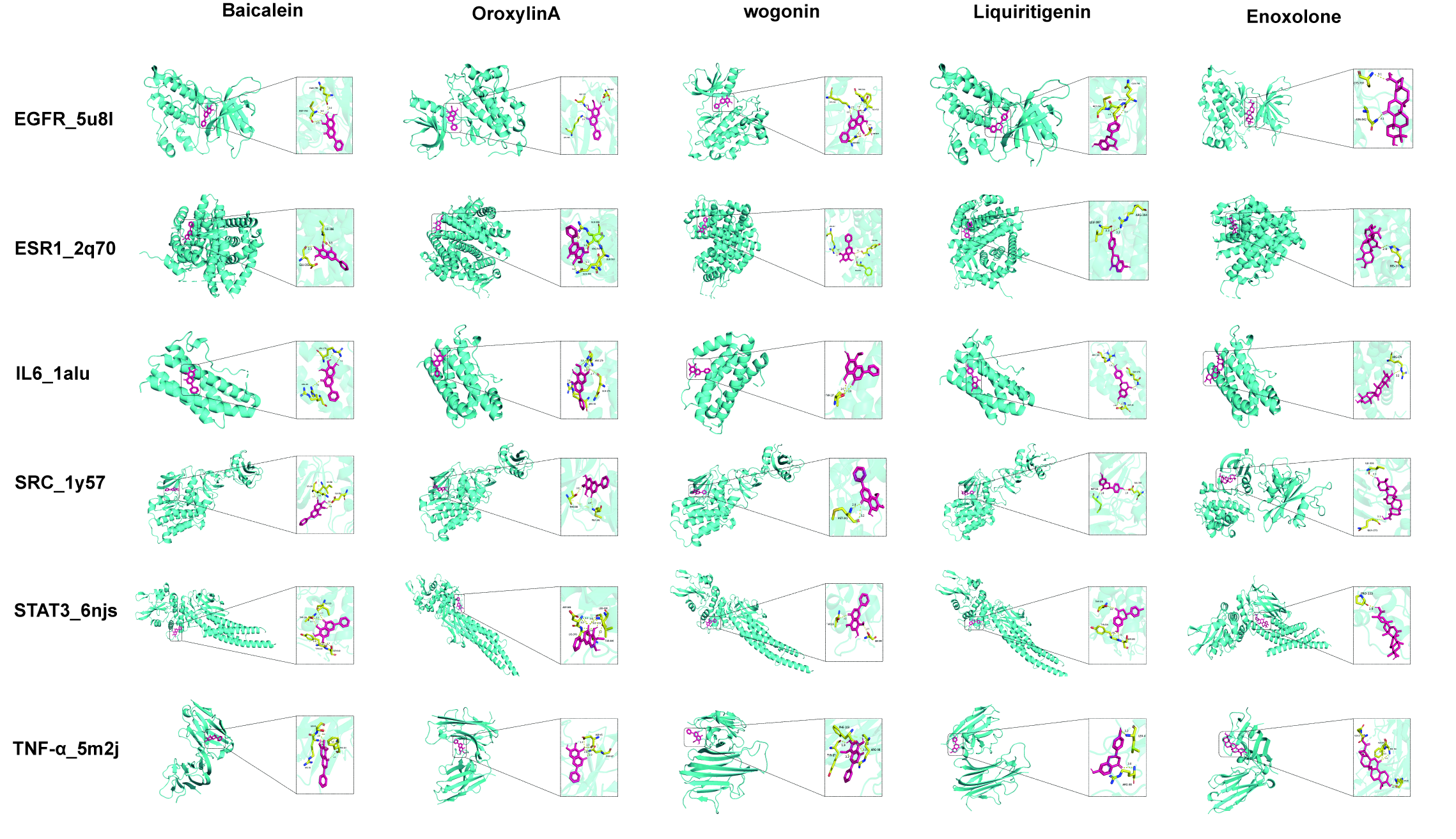

Supplement: Supplementary file 5 [file Image_1.TIF]
